# Symbiotic peptides modulate rhizobial physiology without terminal differentiation

**DOI:** 10.1126/sciadv.aed2816

**Published:** 2026-06-26

**Authors:** Bin Hu, Robert Hänsch, Kyra Grunau, Lena Hohtanz, Martin Kucklick, Nele Grünig, Veit G. Hänsch, Rui Liu, Tong Peng, Max Wiebicke, Ilnaz Sarhangi Fard, Maxim Messerer, Franka Gädeke, Theresia E. B. Stradal, Marius Milatz, Christian Hertweck, Susanne Engelmann, Heinz Rennenberg, Kevin D. Oliphant

**Affiliations:** ^1^ Center of Molecular Ecophysiology (CMEP), College of Resources and Environment, Southwest University, Chongqing 400715, P.R. China.; ^2^ College of Resources and Environment, Southwest University, Chongqing 400715, China.; ^3^ Institute for Plant Biology, Technische Universität Braunschweig, Braunschweig 38106, Germany.; ^4^ Department of Biosciences and Bioengineering, Indian Institute of Technology, Roorkee, Uttarakhand 247667 India.; ^5^ Department of Cell Biology, Helmholtz Centre for Infection Research (HZI), Braunschweig 38124, Germany.; ^6^ Institute for Microbiology, Technische Universität Braunschweig, Braunschweig 38106, Germany.; ^7^ Microbial Proteomics, Helmholtz Centre for Infection Research (HZI), Braunschweig 38124, Germany.; ^8^ Department of Biomolecular Chemistry, Leibniz Institute for Natural Product Research and Infection Biology (HKI), Jena 07745, Germany.; ^9^ Institute of Geomechanics and Geotechnical Engineering, Technische Universität Braunschweig, Braunschweig 38106, Germany.; ^10^ Plant Genome and Systems Biology, Helmholtz Munich, German Research Center for Environmental Health, München-Neuherberg 85764, Germany.; ^11^ Institute for Physical and Theoretical Chemistry, Technische Universität Braunschweig, Braunschweig 38106, Germany.; ^12^ Institute of Microbiology, Faculty of Biological Sciences, Friedrich Schiller University Jena, Jena 07743, Germany.

## Abstract

Symbiotic nitrogen fixation reduces reliance on synthetic fertilizers and is central to agriculture and ecosystem functioning. In legumes, this process occurs in root nodules where rhizobia differentiate into bacteroids that either remain viable or undergo terminal differentiation, a strategy that can enhance nitrogen fixation but limits nodule life span. This irreversible program suits annual legumes and has evolved convergently across multiple lineages, including the inverted repeat-lacking clade (IRLC), where it is enforced by nodule-specific cysteine-rich (NCR) peptides. By contrast, perennial legumes with indeterminate nodules must sustain symbiosis over extended periods, which is incompatible with terminally differentiated bacteroids. Here, we identify a family of nodule-specific proline-glycine-rich peptides (NPGs) in *Robinia* that are induced upon rhizobial infection. NPGs are highly expressed in nodules, encode intrinsically disordered peptides, and accumulate in infected cells within the fixation zone. Exposure of *Mesorhizobium robiniae* to recombinant NPGs induces transcriptional changes associated with a fixation-related physiological state while preserving bacterial viability. These findings identify NPGs as candidate host effectors at the plant-microbe interface and point to an alternative mode of symbiont modulation in perennial legumes.

## Teaser

A previously unidentified peptide family supports nonterminal symbiont modulation in perennial legumes.

## INTRODUCTION

Legume-rhizobia symbioses are a major contributor to global nitrogen fluxes, sustaining ecosystems and agriculture through biological nitrogen fixation (BNF) (*1*–*3*). In root nodules, rhizobia differentiate into bacteroids that either retain reproductive capacity or undergo terminal differentiation, a process marked by cell enlargement, endoreduplication, and loss of viability (*4*–*6*). Nodules occur in two main forms: determinate nodules, in which all infected cells are at a similar developmental stage, and indeterminate nodules, which maintain a persistent meristem and a longitudinal developmental gradient (*7*, *8*). In the inverted repeat-lacking clade (IRLC), which forms indeterminate nodules, terminal differentiation is enforced by nodule-specific cysteine-rich (NCR) peptides, a highly diverse family of defensin-like effectors. Many NCRs are cationic and profoundly reprogram bacterial physiology, promoting nitrogen fixation at the cost of symbiont viability (*6*, *9*–*11*).

By contrast, Robinioid legumes, the sister clade of the IRLC, develop indeterminate nodules but lack NCR peptides (*12*). In these taxa, bacteroids generally remain reproductively competent, and nitrogen fixation can be sustained across multiple growing seasons (*13*–*15*). The Robinioid tree *Robinia pseudoacacia* exemplifies this strategy, maintaining active nodules for extended periods in nutrient-poor and disturbed soils and contributing to reforestation, land reclamation, and phytoremediation efforts (*15*–*19*). As the sole sister lineage to the NCR-bearing IRLC within the Hologalegina, Robinioids occupy a pivotal phylogenetic position for examining alternative strategies of host control in legume symbiosis.

This contrast highlights an unresolved question in legume biology: How do perennial legumes with indeterminate nodules bias their symbionts toward nitrogen fixation without resorting to terminal bacteroid differentiation? Comparative studies indicate that nodulation in *R. pseudoacacia* involves systemic transcriptional reprogramming and developmental trajectories distinct from those described in herbaceous model legumes (*14*). However, the identity of host-derived factors acting directly at the plant-microbe interface in Robinioids has remained unclear.

Beyond NCRs, legumes deploy a diverse repertoire of host-derived molecules to regulate symbiosis, including defensin-like peptides, CLAVATA3/ESR-related (CLE) peptides (*20*), transcriptional regulators such as Nodule Inception (NIN), Early Nodulins (ENODs), and Nuclear Factor-Y (NF-Y) subunits (*21*). In addition, several classes of glycine-rich proteins have been reported to accumulate in nodules or nodulated roots, although their functions and modes of action remain poorly characterized (*22*–*24*). Together, these examples illustrate that symbiont control is achieved through multiple molecular strategies, many of which remain incompletely understood outside a small number of model systems.

Here, we identify a family of nodule-specific proline-glycine-rich peptides (NPGs) in *R. pseudoacacia*. These peptides are highly expressed in nodules, encode intrinsically disordered peptides, and localize preferentially to infected host cells. Exposure of *Mesorhizobium robiniae* to recombinant NPGs induces transcriptional changes associated with a nitrogen fixation-related physiological state while preserving bacterial viability. Rather than enforcing terminal differentiation, NPGs therefore represent a candidate class of host-derived peptides that may modulate symbiont physiology in a nonlethal manner. This work expands the known diversity of nodule-associated peptides and highlights an alternative mode of host-microbe interaction in perennial legumes.

## RESULTS

### NPGs define a previously unknown family of nodule-specific peptides

**Fig. 1.**
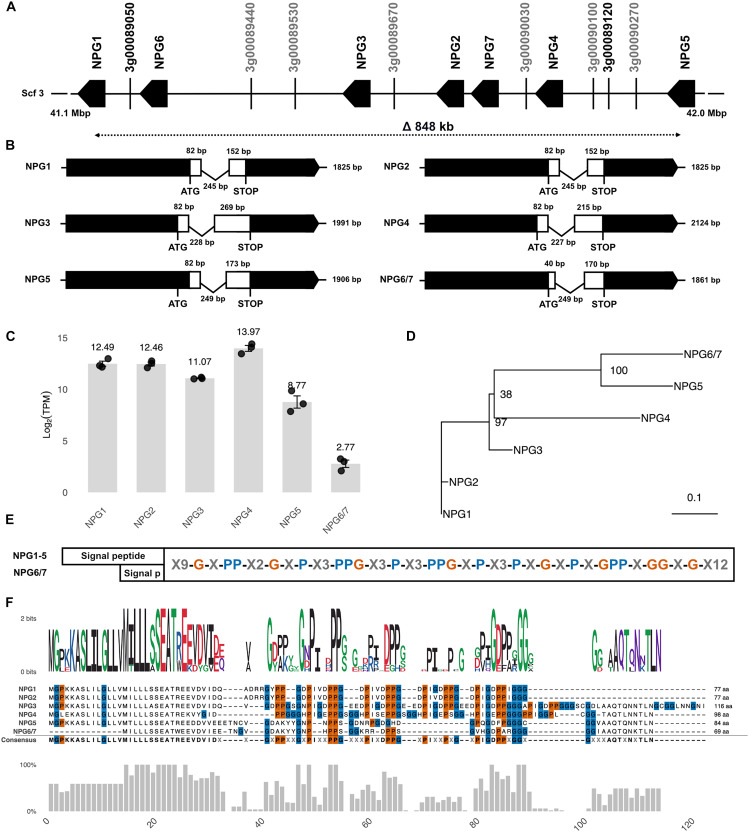
Genomic organization, transcript architecture, and evolutionary description of NPG genes. (**A**) Genomic context of the NPG gene cluster on scaffold 3. Nodule-specific genes are shown in black, non-nodule–specific genes in gray. Arrows indicate transcriptional orientation. (**B**) Transcript structures of NPG paralogs reconstructed from Oxford Nanopore Technologies (ONT) long-read RNA-seq data. Exons are shown as black boxes, introns are shown as thin lines, and CDSs are shown as white boxes. (**C**) Expression levels of NPG genes in 1-year-old nodules postinoculation, shown as log_2_(TPM). Individual replicates (*n* = 3) are overlaid as dots; means ± SD. (**D**) Unrooted maximum-likelihood phylogenetic tree of full-length NPGs inferred in IQ-TREE under the JTT model. Branch lengths reflect evolutionary distance. Ultrafast bootstrap values > 50% (*n* = 1000) are indicated. (**E**) Amino acid composition of NPG peptides showing conserved glycine (brown) and proline (blue) residues within the mature region. (**F**) Sequence conservation across NPGs shown as the (top) sequence logo based on information content (bits), colored by residue chemistry; (middle) MAFFT alignment with Pro and Gly highlighted; and (bottom) residue-wise conservation scores calculated by Valdar’s method. aa, amino acids.

RNA sequencing (RNA-seq) of *R. pseudoacacia* nodules inoculated with *M. robiniae* uncovered seven transcripts encoding NPGs peptides, here termed *NPG*s (table S1). These genes occur as a tandem cluster within an ~850-kb region, consistent with duplication (Fig. 1A) (*25*, *26*). All seven *NPG*s were highly expressed in nodules 1 year after inoculation, some ranking just below leghemoglobins among the most abundant transcripts (Fig. 1, B and C).

Long-read sequencing confirmed conserved intron-exon structures and shared regulatory features across paralogs (Fig. 1B). Phylogenetic analysis grouped NPG1/NPG2 as near-identical, whereas NPG5 and the identical NPG6/NPG7 formed the most divergent branch (Fig. 1D). Most members carried canonical signal peptides, although NPG6/NPG7 encode truncated motifs (Fig. 1, E and F, and fig. S1). Proteomic analysis of nodule extracts confirmed translation of *NPG2* and *NPG3*, with high-confidence peptide-spectrum matches for multiple peptide fragments (fig. S2 and table S2). Although NPG1 and NPG2 share several peptide sequences, protease digestion mapping and mass spectrometry (MS) analysis identified peptides unique to NPG2, allowing unambiguous discrimination of this paralog (fig. S3). In contrast, NPG3 yielded a distinct set of unique peptides, confirming translation of this divergent family member. The remaining paralogs were not detected in proteomic datasets, likely reflecting low abundance and/or technical challenges associated with recovering intrinsically disordered, low-complexity peptides by MS. These results identify NPGs as a small, conserved orthogroup of nodule-specific peptides (69 to 116 amino acids). Automated annotation assigned NPG5 to a generic glycine-rich protein category based on low-complexity sequence composition; however, NPGs lack conserved domains characteristic of previously described glycine-rich nodulins or defense-associated proteins, and this annotation likely reflects compositional bias rather than functional relatedness (*27*).

### Nodule-specific induction and spatial restriction define *NPG* gene expression

**Fig. 2.**
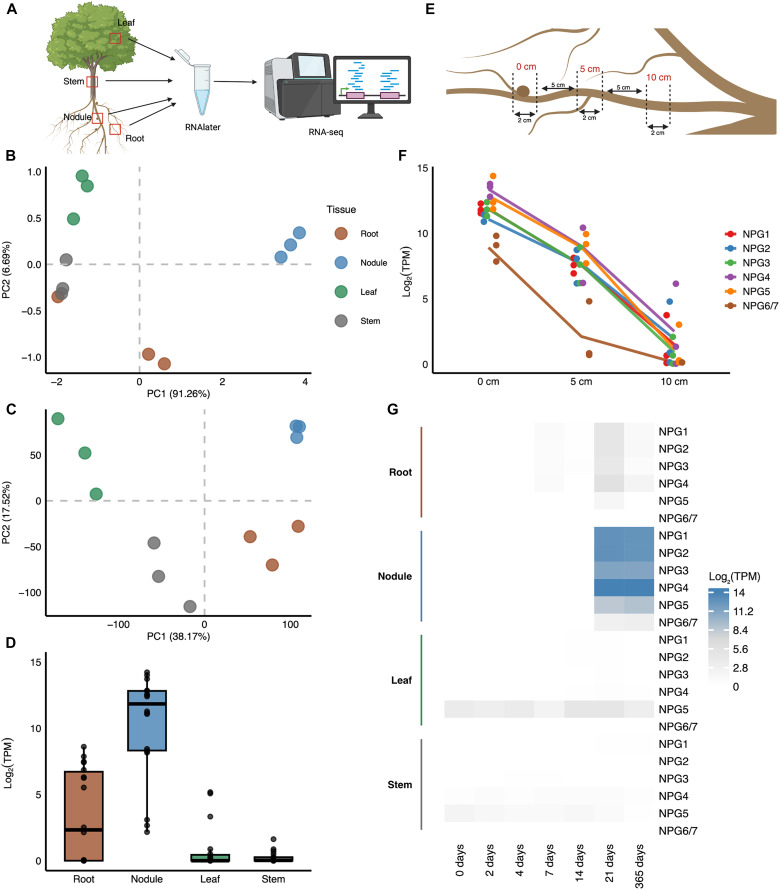
NPG gene expression is nodule-specific with distinct spatial and temporal patterns. (**A**) Sampling strategy for transcriptomic profiling at multiple time points postinoculation, including leaves, stems, roots, and nodules. (**B**) PCA of NPG expression at 21 dpi based on log_2_(TPM). PC1 (91.26%) and PC2 (6.69%) explain 97.95% of variance. Each point represents one plant, colored by tissue (brown: root; blue: nodule; green: leaf; and gray: stem). (**C**) PCA of global expression (30,295 genes) from the same dataset; PC1 and PC2 explain 38.17 and 17.52% of variance, respectively. (**D**) Boxplot of NPG expression across tissues at 21 dpi [log_2_(TPM)]; points are individual NPG genes per replicate. Boxes show the interquartile range; lines indicate the median. (**E**) Root dissection scheme for spatial expression profiling, with samples from nodules (0 cm) and root sections 5 and 10 cm downstream. (**F**) Expression of individual NPG genes across root sections at 30 dpi [log_2_(TPM)]; points show replicates, and lines indicate regression. (**G**) Heatmap of NPG expression across tissues and time points [log_2_(TPM)]; values are means from three replicates. Color scale: gray (low) to blue (high); no clustering applied. [(A) and (E)] Created in BioRender, https://BioRender.com/g2xgoka.

To resolve *NPG* expression dynamics, we profiled *R. pseudoacacia* plants inoculated with *M. robiniae* by RNA-seq across roots, nodules, stems, and leaves at multiple time points following inoculation (Fig. 2A). Computed tomography (CT) imaging was used to nondestructively monitor nodule emergence and early growth in intact root systems and to determine when nodules reached a size compatible with reliable physical isolation. CT analyses showed that nodules first became detectable ~3 weeks postinoculation; before this point, nodules were absent or too small to be harvested for transcriptomic analysis (fig. S4 and movies S1 to S4). Accordingly, early RNA-seq time points necessarily sampled whole roots rather than isolated nodules, capturing prenodulation and early symbiotic stages. Once formed, nodules expanded compactly over subsequent months, with no evidence of rapid structural decline, indicating sustained post–emergence growth. This contrasts with indeterminate nodules of IRLC model legumes, which typically enter senescence within weeks after nodulation (*28*).

Principal components analysis (PCA) of all expressed transcripts separated nodules from other organs, reflecting global transcriptional differences between tissues. PCA restricted to *NPG* transcripts similarly resolved nodules from non-nodule tissues (Fig. 2, B and C), indicating that *NPG* expression is strongly biased toward nodules and reflects underlying organ-specific transcriptional states.

Quantitative profiling showed that *NPG* expression is strongly enriched in nodules, with substantially lower levels detected in adjacent root tissue and negligible signal in stems and leaves (Fig. 2D). Along the root axis, *NPG6*/*NPG7* expression declined sharply outside nodules, whereas *NPG1*-*NPG5* exhibited low but detectable expression several centimeters from the nodule (Fig. 2, E and F). No *NPG* transcripts were detected in roots of uninoculated plants, indicating that *NPG* expression is induced by nodulation and is tightly associated with nodulated root regions rather than with roots per se.

Temporal analysis revealed that *NPG* transcripts were first detectable only after nodules had formed and reached a harvestable size, ~3 weeks postinoculation, and remained elevated for at least 1 year thereafter (Fig. 2G). No increase in *NPG* expression was observed in roots during earlier nodulation stages before visible nodule formation. Among the paralogs, *NPG4* was the most abundant transcript, followed by *NPG1*/*NPG2*; *NPG3* remained low, *NPG5* increased during later maturation, and *NPG6*/*NPG7* were minimal throughout. These results indicate that *NPG* expression is induced after nodule formation and is associated with established nodules rather than with early nodulation or prenodulation developmental processes.

### NPG signal peptides mediate secretion and reflect functional divergence within the family

To test whether NPGs enter the secretory pathway, we examined their signal peptides and biochemical features. DeepLoc2.1 predicted all NPG paralogs to be secreted, consistent with the presence of N-terminal signal peptides (fig. S1 and table S3). NPG1 to NPG5 encoded a conserved 25–amino acid signal peptide, whereas NPG6 and NPG7 carry a truncated 9–amino acid motif. These predictions indicate targeting to the secretory pathway but do not resolve the final destination of the peptides, such as secretion into the general apoplast or delivery to specialized compartments at the plant-microbe interface.

**Fig. 3.**
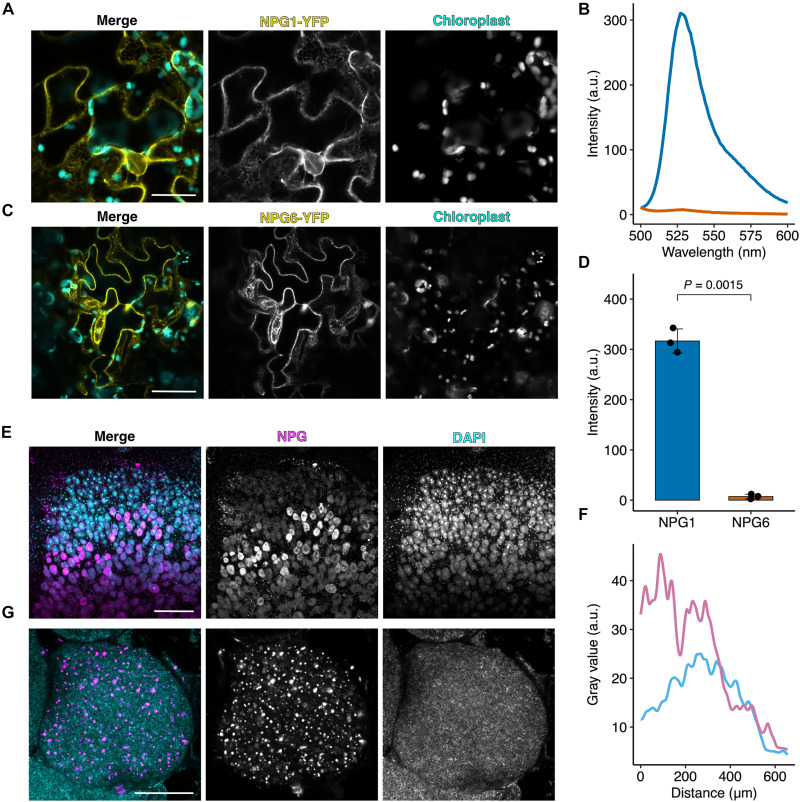
NPGs display punctate localization in nitrogen-fixing nodule cells. (**A** and **C**) Confocal images of *N. benthamiana* leaves transiently expressing NPG1-YFP (A) or NPG6-YFP (B) under the CaMV 35*S* promoter. YFP in yellow; chloroplast autofluorescence in cyan. a.u., arbitrary units. Scale bars, 50 μm. (**B**) Emission spectra of YFP fluorescence in AWF from (A) and (C), excited at 488 nm. Black: NPG1-YFP; red: NPG6-YFP. Means of three biological replicates. (**D**) Quantification of the YFP signal in AWF from three independent infiltrations. Bars show means ± SD. Color code as in (B). (**E**) Cryosection (20 μm) of an indeterminate *R. pseudoacacia* nodule showing the proximal (rootward) bottom and the distal apical meristem at the top, with intervening infection and central nitrogen-fixation zones (*38*). NPG immunofluorescence in magenta; DAPI-stained rhizobia in cyan. Scale bar, 200 μm. (**F**) Line plot of normalized gray values along the nodule axis from base to apex, indicating the zonal distribution of NPG signal. (**G**) High-magnification view of an infected nodule cell. NPG immunosignal (magenta) forms puncta that do not colocalize with DAPI-stained rhizobia (cyan). Scale bar, 20 μm.

To assess whether NPG signal peptides are sufficient to direct proteins into the secretory pathway, we cloned the full genomic sequences of NPG1 and NPG6 (exon 1–intron–exon 2) in-frame with yellow fluorescent protein (YFP), generating C-terminal NPG-YFP fusions. Under the CaMV 35*S* promoter, these constructs were transiently expressed in *Nicotiana benthamiana*. Confocal imaging 4 days after infiltration showed that NPG1-YFP accumulated in the apoplast, whereas NPG6-YFP remained intracellular (Fig. 3, A and C). Analysis of apoplastic wash fluid (AWF), collected from *N. benthamiana* leaves, corroborated secretion of NPG1-YFP but not NPG6-YFP (Fig. 3B). These results demonstrate that the full-length signal peptides of NPG1 to NPG5 are sufficient to confer secretion competence, whereas the truncated motifs of NPG6/NPG7 are not. This heterologous assay confirms the functionality of NPG signal peptides in directing proteins into the secretory pathway, whereas localization to specialized compartments within *R. pseudoacacia* nodules remains to be resolved in the native symbiotic context.

### NPGs exhibit modular intrinsic disorder and conserved repeat architecture

Although *NPG4* was the most abundant transcript, MS provided strong, reproducible peptide evidence only for NPG2 (table S2). NPG1 could not be distinguished from NPG2 due to sequence overlap, and NPG3 yielded unique peptides only sporadically and at low intensity. We therefore selected NPG2 for biochemical and functional analysis as it yielded the most robust and reproducible peptide evidence by MS.

To explore structural properties, we modeled the seven NPG paralogs with AlphaFold3 (*29*) (fig. S5). All showed uniformly low predicted local distance difference test (pLDDT) scores (<70) across mature peptide regions, consistent with high intrinsic disorder and the absence of reliably predicted tertiary structure (*30*, *31*). As representative examples, NPG1 and NPG2 displayed a modular organization comprising an acidic N-terminal segment followed by tandem proline-glycine-rich repeat motifs (tables S4 and S5). These repeat units are conserved in sequence composition, length, and register across paralogs and are encoded by nonrandom codon patterns, indicating that their organization has been preserved by selective constraint rather than arising solely from neutral repeat expansion (*32*). NPG1 contains two consecutive PPGDPIVD motifs followed by two PPGDPIGD variants; NPG2 carries three uninterrupted PPGDPIVD motifs and one PPGDPIGD, indicating enhanced repeat regularity that may support multivalent or low-affinity interactions.

To test these predictions, we synthesized short NPG2-derived peptides representing the acidic N-terminal motif, a basic linker, a single repeat, tandem repeats, and a composite acidic-basic fragment (table S5). All peptides were N-terminally acetylated and C-terminally amidated to approximate native processing. Liquid chromatography–mass spectrometry (LC-MS) detected exclusively single-mass species for all variants under the conditions tested, with no evidence of higher-mass assemblies (fig. S6, A and B). Complementary two-dimensional (2D) nuclear magnetic resonance (NMR) experiments supported this interpretation: nuclear Overhauser effect spectroscopy (NOESY) spectra showed no long-range cross-peaks beyond those present in correlation spectroscopy (COSY) spectra, consistent with a lack of stable tertiary interactions and behavior expected for intrinsically disordered peptides (fig. S7). CD spectroscopy further indicated predominantly random coil conformations, with a pronounced minimum near 200 nm and no detectable α-helical or β sheet content (fig. S6, C and D). Together, these analyses indicate that NPG-derived peptides behave as intrinsically disordered, monomeric species under the conditions examined, with modular repeat architecture and high structural flexibility.

### NPGs accumulate in a subset of infected nodule cells in the nitrogen fixation zone

Immunofluorescence microscopy using affinity-purified polyclonal antibodies against recombinant NPG2 revealed a strong punctate signal confined to large, infection-associated cells in the central zone of *R. pseudoacacia* nodules. These cells contained numerous 4′,6-diamidino-2-phenylindole (DAPI)–stained rhizobia but lacked the hypertrophied, endoreduplicated bacteroids characteristic of *Medicago truncatula* and other IRLC legumes (*5*, *33*). Signal was strongest in infected cells, forming a gradient from the nodule center toward the periphery, with only faint apoplastic signal and no detection in uninfected cells or other tissues (Fig. 3, E and F). The peptides localized to discrete cytoplasmic foci, frequently adjacent to, but not overlapping with, rhizobial DNA, and were not observed within bacterial cells (Fig. 3G). Omission of the primary antibody abolished staining (fig. S8), confirming specificity. Given the high sequence similarity among paralogs, these signals are most conservatively interpreted as representing the NPG family as a whole rather than a single isoform.

In contrast to the predominantly apoplastic localization observed in transient expression assays in *N. benthamiana*, NPGs in *R. pseudoacacia* nodules were confined to infected host cells and accumulated in discrete intracellular foci proximal to rhizobia. This localization pattern is consistent with, but does not demonstrate, association with symbiotic compartments and suggests that NPG deployment is coupled to infection status (*34*).

### Exposure to NPG2 alters rhizobial gene expression

RNA-seq of *M. robiniae* cultures exposed to recombinant NPG2 (250 μM) or phosphate-buffered saline (PBS) controls were used to probe the transcriptional responsiveness of the bacterium. This concentration was selected to define the ceiling of transcriptional responsiveness. Sequencing produced high-quality libraries (Q30 > 91.9%; >91% mapping; >97% gene body coverage), with strong replicate correlation (≥0.94) and clear PCA separation (fig. S9A). Differential expression analysis identified 1022 loci with altered transcript abundance [false discovery rate (FDR) < 0.05; |log_2_ fold change| ≥ 1] following NPG2 exposure (fig. S9B).

**Fig. 4.**
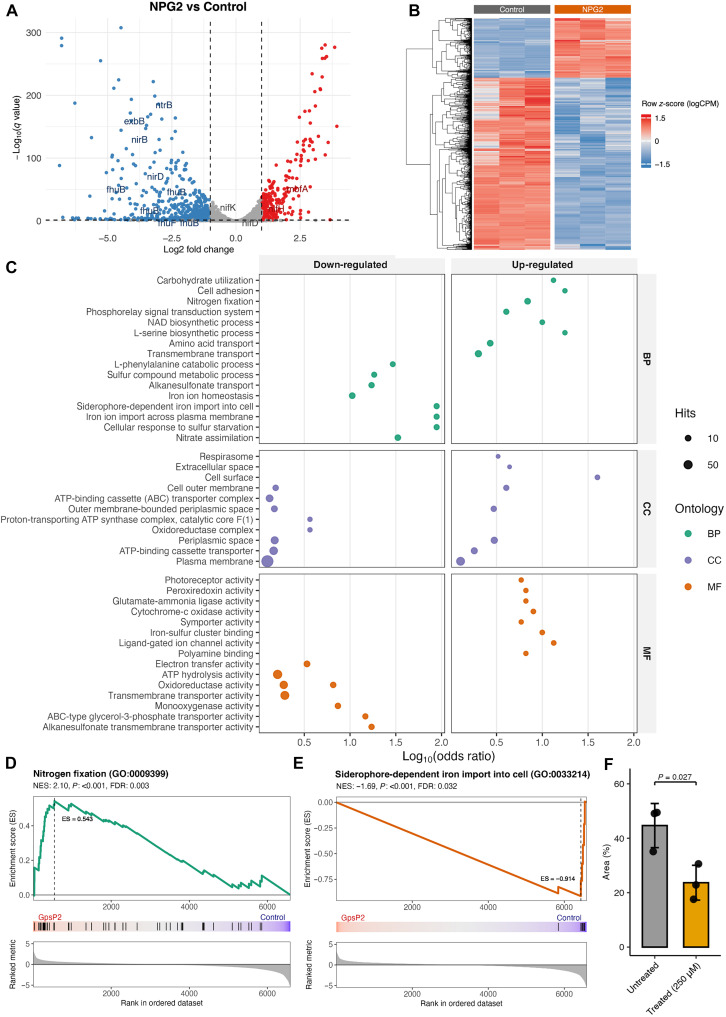
NPG2 induces a fixation-associated transcriptional program in *M. robiniae*. (**A**) Volcano plot of DEGs after 2-hour exposure to 250 μM NPG2. Dashed lines mark |log_2_ fold change| ≥ 1 and *q* ≤ 0.05. Selected genes related to nitrogen fixation (e.g., *nifH*, *nifD*, and *nifK*) and iron/Fe-S metabolism (*mbfA*, *fhuB*, *fhuF*, *exbB*, *nirB*, *nirD*, and *ntrB*) are highlighted. (**B**) Heatmap of DEGs across replicates; values are row-scaled logCPM (*z*-scores). Rows are clustered, and columns are grouped by condition. (**C**) GO enrichment of DEGs. Point size indicates the gene number; color denotes the ontology (BP, CC, and MF); the *x* axis shows log_10_(odds ratio). (**D** and **E**) GSEA plots for representative pathways. Curves show running enrichment score (ES); ticks mark gene positions. Normalized enrichment score (NES), nominal *P*, and FDR are shown. Gradient bars rank genes from NPG2-associated (left) to control (right). (**F**) Growth assay of *M. robiniae* treated for 2 hours with 250 μM NPG2 or untreated control and then plated on YEM agar. Bars show the mean colony area ± SD (*n* = 3); points indicate replicate means.

Induced transcripts prominently included *nifHDK* and accessory nitrogenase genes, together with transporters and the iron storage protein *mbfA*, whereas genes involved in nitrate reduction, siderophore uptake, and nitrogen regulation (*nirBD*, *fhuB/F*, *exbB*, and *ntrB*) were strongly repressed (Fig. 4, A and B, and fig. S9C). Functional enrichment analysis indicated coordinated activation of nitrogen fixation, iron homeostasis, and amino acid biosynthesis pathways, accompanied by repression of nitrate assimilation, siderophore import, and sulfur-associated responses (Fig. 4C and fig. S9D). Together, these results indicate that exposure to NPG2 is sufficient to elicit a structured and pathway-coherent transcriptional response in *M. robiniae* that favors nitrogen fixation-associated programs over alternative nitrogen acquisition strategies.

### NPGs bias rhizobial transcription toward a fixation-associated state without loss of viability

Gene set enrichment analysis (GSEA) of transcripts induced following NPG2 treatment revealed significant enrichment of nitrogen fixation genes, including nitrogenase structural and accessory loci (Fig. 4D). Genes associated with nitrate assimilation were correspondingly depleted, consistent with the reciprocal regulation of nitrogen fixation and nitrate use in rhizobia (*35*). GSEA further showed strong negative enrichment of siderophore-dependent iron import pathways (Fig. 4E), reflecting coordinated repression of outer membrane and periplasmic iron uptake systems (*36*).

To assess sensitivity and specificity, transcript levels of representative NPG2-responsive genes were quantified by quantitative polymerase chain reaction (qPCR) across a concentration series. NPG2 elicited a dose-dependent transcriptional response in *M. robiniae*. Induction of the nitrogenase gene *nifH* was detectable at 0.025 μM and increased monotonically with peptide concentration, reaching an ~3-fold up-regulation at 250 μM (fig. S10A). In contrast, *mbfA* expression remained unchanged at submicromolar concentrations and was robustly induced only at 250 μM. The nitrate reductase gene nirB exhibited a biphasic response, with modest, nonsignificant induction at low micromolar concentrations and mild repression at 250 μM. Truncated peptides corresponding to either the N-terminal or repeat region failed to recapitulate this transcriptional profile even at 250 μM, demonstrating that full-length NPG2 is required for activity (fig. S10B).

Exposure of log-phase *M. robiniae* to recombinant NPG2 reduced subsequent colony expansion in a dose-dependent manner, whereas an inactive thioredoxin A (TrxA)–NPG2 fusion protein had no detectable effect (Fig. 4F and fig. S11). Colony formation was retained, indicating preserved viability. Together, these results indicate that NPG exposure biases rhizobial physiology toward a fixation-associated transcriptional state while constraining bacterial expansion without inducing bactericidal effects.

### NPG peptides are a conserved, genus-wide family unique to *Robinia*

To test whether NPGs occur beyond *R. pseudoacacia*, we searched the National Center for Biotechnology Information (NCBI) nonredundant nucleotide (nt) and protein (nr) databases (release July 2025). No homologs were detected, consistent with the absence of annotated *Robinia* genomes in these repositories. The first published *R. pseudoacacia* genome assembly likewise failed to annotate *NPG*s as conventional ab initio and homology-based pipelines overlooked these small, compositionally biased loci despite their presence in the assembly. By contrast, our *R. pseudoacacia* reference genome, informed by RNA-seq, long-read, and proteomic evidence, captured the full *NPG* complement.

Surveying over 400 Fabaceae genomes currently deposited at the NCBI, including ~20 tree species, revealed no homologs outside Papilionoideae and only low-similarity short motifs within that subfamily (fig. S12). Phylogenetic placement based on the recently resolved papilionoid tree shows *R. pseudoacacia* as the only sequenced woody species within Hologalegina (fig. S13). At present, no woody perennial genome from either the Robinioids or IRLC is available, leaving broader distribution unresolved.

To assess conservation, we sequenced four additional *Robinia* lineages (*R. neomexicana*, *R. hispida* var. *kelseyi*, *R. viscosa*, and *R. hispida*). All encoded the full paralog *NPG* set found in *R. pseudoacacia*, with intact coding sequences (CDSs) and no evidence of pseudogenization (fig. S14). Together, these results establish NPGs as a conserved, genus-wide peptide family of *Robinia*.

## DISCUSSION

We identify a previously undescribed family of small, acidic NPGs that are conserved across *Robinia* but absent from other sequenced legumes. Their genus-wide conservation, together with the absence of NCR peptides from woody Robinioid lineages, highlights a pronounced evolutionary contrast within the Hologalegina, in which closely related clades appear to have adopted distinct molecular strategies for regulating symbiotic bacteria. Whereas IRLC legumes deploy large repertoires of NCR peptides that irreversibly enforce bacteroid differentiation, *Robinia* encodes a compact yet highly expressed family of NPGs that modulate rhizobial physiology without inducing terminal fate.

Although our genomic survey of ~120 Papilionoideae assemblies suggests that NPGs are confined to *Robinia*, woody legumes remain underrepresented in public genome databases. It therefore remains possible that related peptide families occur more broadly but have escaped detection due to incomplete sampling and the annotation challenges associated with small, compositionally biased genes. Sequencing additional Robinioid species and other woody members of the Hologalegina will be necessary to resolve the evolutionary distribution of this peptide family.

NPGs differ from NCRs in both sequence composition and apparent mode of action. NCRs are short, cysteine-rich, defensin-like peptides that impose terminal differentiation (*6*, *11*), whereas NPGs are intrinsically disordered, acidic peptides that accumulate preferentially in infected host cells and are not associated with loss of bacterial viability. Their heterogeneous localization across infected cells parallels evidence from single-cell transcriptomic studies, indicating that nodule tissues are functionally heterogeneous rather than uniform (*37*–*39*). In contrast to NCRs, which peak early in the interzone, NPGs display a broader and patchier distribution within the fixation zone and beyond, consistent with cell type–specific deployment rather than uniform enforcement of a single differentiation program.

Glycine-rich proteins and peptides have previously been reported in legume nodules and nodulated roots, most commonly as glycine-rich nodulins or nodule-induced glycine-rich proteins identified through expression profiling and spatial localization analyses. These proteins are typically substantially larger than NPGs, frequently exceeding several hundred amino acids, and often comprise extended glycine-rich regions embedded within otherwise structured domains (*23*). In most cases, they lack clear signal peptides, show no specific enrichment at the symbiotic interface, and have been inferred to function in cell wall organization, RNA binding, or general developmental and stress-associated processes rather than in direct modulation of bacteroid physiology. Accordingly, evidence for their involvement in symbiont control has remained largely indirect (*23*, *40*). In this respect, NPGs resemble NCR peptides more closely than previously described glycine-rich nodulins: Like NCRs, they are short, nodule-specific peptides transcriptionally expressed at exceptionally high levels and capable of eliciting coordinated transcriptional responses in rhizobia. However, NPGs do not conform to any previously described class of nodule-associated peptides or glycine-rich proteins, lacking the structural features and functional signatures that define established nodule pathways described to date. Instead, they constitute a distinct class of host-derived peptides that modulate symbiont physiology.

Despite comprising only seven paralogs, NPGs rank among the most abundant transcripts in *R. pseudoacacia* nodules, approaching the expression levels reported for NCR peptides in *M. truncatula* (*41*). This high apparent dosage, achieved through transcript abundance rather than extensive family expansion, may reflect a requirement for continuous synthesis of unstable, intrinsically disordered peptides to sustain biologically effective concentrations (*42*). Functional diversification within the family appears to occur through differences in targeting, with full-length signal peptides mediating secretion and truncated variants remaining intracellular.

The bacterial response to recombinant NPG2 further distinguishes NPGs from known nodule peptides. Transcriptomic profiling of *M. robiniae* exposed to NPG2 revealed reproducible induction of nitrogenase structural and accessory genes together with changes in iron-associated functions, whereas pathways associated with nitrate assimilation, siderophore uptake, and quorum sensing were repressed. These transcriptional responses occurred without detectable stress signatures or loss of bacterial viability, indicating that NPG exposure biases rhizobial gene expression toward a fixation-associated physiological state rather than exerting bactericidal or terminally differentiating effects (*43*, *44*). The experiments used a fixed concentration of 250 μM recombinant NPG2 to define the maximal transcriptional response of the bacterium. The endogenous concentration of NPGs in nodules has not yet been determined, and the applied concentration may exceed physiological levels. However, dose-response analyses (fig. S10) show that *nifH* induction is detectable at concentrations as low as 0.025 μM and increases monotonically with peptide concentration, indicating activity across a broad concentration range. These observations support the biological relevance of NPG2 activity while also indicating that the transcriptional profiles obtained at 250 μM reflect a maximal-response condition rather than directly mirroring the in planta environment. Given that NPG transcripts account for a substantial fraction of total nodule mRNA, their abundance is consistent with the magnitude of the transcriptional changes observed, although direct effects on nitrogen fixation activity in planta remain to be demonstrated. Quantitative measurements of NPG abundance in nodules will be required to establish physiologically relevant concentration ranges.

From an evolutionary perspective, NPGs illustrate how legume peptide repertoires may diversify along distinct trajectories. In herbaceous IRLC legumes, NCR peptides enforce terminal differentiation in line with annual life histories, whereas in woody *Robinia*, NPGs are associated with modulation of rhizobial physiology without loss of viability. This distinction is consistent with, but does not by itself demonstrate, an association between peptide-mediated control strategies and perennial nodulation. Even within Robinioids, divergent solutions appear to exist: *Lotus japonicus* lacks both NCRs and NPGs and instead relies on apoplastic diffusion barriers to restrict rhizobial spread (*45*). Together, these examples underscore that symbiont control is not governed by a universal mechanism but is shaped by lineage-specific innovations and ecological context.

Ecologically, the presence of NPGs may contribute to the capacity of *R. pseudoacacia* to function as a pioneer species in degraded, nutrient-poor environments. By maintaining viable symbionts while biasing their physiology toward nitrogen fixation, NPGs provide a plausible molecular component of the long-term persistence of perennial nodules, although additional genetic and physiological evidence will be required to substantiate this link. At the ecosystem scale, such mechanisms could help explain the documented contributions of *R. pseudoacacia* to soil enrichment, forest regeneration, and successional dynamics.

Beyond their evolutionary importance, NPGs suggest alternative directions for applied strategies aimed at improving symbiotic nitrogen fixation. Many peptide-based approaches have focused on NCR-like or antimicrobial activities, which inherently risk compromising rhizobial survival. By contrast, NPGs illustrate how host-derived peptides can influence symbiont gene expression without enforcing terminal differentiation, highlighting a potentially tractable but as yet untested avenue for engineering symbiotic compatibility.

Several questions remain unresolved. The bacterial receptors or molecular targets of NPGs are unknown, as are the functional distinctions among paralogs and the mechanisms underlying their heterogeneous accumulation within nodules. Genetic disruption of NPG loci in *R. pseudoacacia* will be essential to assess their contribution to symbiotic performance in planta.

Comparative analyses across additional perennial legumes will clarify whether NPGs represent a *Robinia*-specific peptide family or a broader feature of woody symbioses. Last, integration of single-cell and compartment-resolved transcriptomics with bacterial genetics may reveal how NPG-derived signals are perceived and translated at the plant-microbe interface.

Overall, the emergence of NPGs exemplifies lineage-specific peptide families in legume symbiosis, consistent with recurrent co-option of host defense and cellular machinery to regulate microbial partners (*46*). Rather than deriving from NCR precursors, NPGs constitute a distinct peptide lineage that is associated with nonlethal modulation of rhizobial physiology. In this sense, they offer one possible answer to the question posed by Downie and Kondorosi: Why do some legumes rely on NCR-driven terminal differentiation, whereas others lack NCRs altogether? Our findings place NPGs within the expanding repertoire of legume peptides that influence symbiosis and underscore that peptide-mediated control of rhizobia has evolved through multiple, chemically distinct solutions.

## MATERIALS AND METHODS

### Plant material and growth conditions

Seeds of *R. pseudoacacia* DB214 provenance were purchased in October 2013 from a commercial tree nursery in Fushun City, Liaoning Province, China (41°51′N, 123°56′E), and transferred to laboratories in Chongqing, P.R. China, and Braunschweig, Germany. Plantation sites near Fushun have a temperate humid to semihumid continental monsoon climate, with mean annual precipitation of ~800 mm (*47*). Topsoil (0 to 10 cm) characteristics were as follows: field water capacity: 27.1%; total N: 1.8 g kg^−1^; total P: 0.65 g kg^−1^; and organic C: 41.3 g kg^−1^ (*48*). The leaf material of *R. neomexicana*, *R. hispida* var. *kelseyi*, *R. viscosa*, and *R. hispida* was obtained from the Botanical Garden Göttingen (Germany).

### Seed sterilization and in vitro propagation

Seeds were scarified in concentrated H_2_SO_4_ for 20 min, rinsed six times in distilled water, surface-sterilized with 6% NaClO (0.025% Tween 20) for 15 min, and rinsed in sterile water. Germination was on solid MS medium at 23°C, 16-hour photoperiod (120 μE m^−2^ s^−1^). Seven-day-old seedlings were transferred to culture tubes for 2 weeks and then to culture jars for 4 weeks. Shoot cuttings with one leaf were subcultured every 4 weeks for 4 months. Seventy-two uniform plantlets were transplanted to 20 cm–by–30 cm pots containing 3 kg of sterilized sand-vermiculite mix [95:5, v/v; water-holding capacity: 15.33% (*49*)].

### Rhizobial strain and inoculation

*M. robiniae* (DSM 100022) originally isolated from root nodules of *R. pseudoacacia* in Yangling, Shaanxi Province, China was obtained from the Leibniz Institute DSMZ-Germany Collection of Microorganisms and Cell Cultures (Braunschweig, Germany). Long-term cultures cultivated in tryptone-yeast extract (TY) were stored as 20% (v/v) glycerol stocks at −80°C. For inoculation, the strain was revived on yeast-mannitol agar (YMA) medium (*50*), transferred to TY broth, and grown at 28°C for 48 hours with shaking (180 rpm). Cultures were centrifuged (2000*g*, 10 min, 25°C) and resuspended in sterile water to OD_600_ (optical density at 600 nm) = 1.0. Roots of 40-day-old seedlings were inoculated with 1 ml of the suspension at the root zone. Eighteen pots (*n* = 3 biological replicates) were inoculated, and an equal number were mock treated with sterile water.

### Nodule monitoring

In vitro–propagated *R. pseudoacacia* plantlets were transferred into rigid polypropylene homopolymer pots (inner diameter: 68 mm; height: 100 mm; and wall thickness: 2 mm) custom-built at the Institute of Geomechanics and Geotechnical Engineering (TU Braunschweig). Each pot contained five drainage openings at the base and was filled with uniform quartz sand (0.63 to 2.0 mm; mean grain diameter *d*_50_ = 0.76 mm). Plants were subirrigated daily by placing the pots in trays, with volumes adjusted so that trays were dry after ~24 hours and maintained under long-day conditions (16-hour light/8-hour dark, ~150 μmol m^−2^ s^−1^, and 20° to 22°C). On day 0, saplings were inoculated with Rhizobium by pipetting 1 ml of the bacterial suspension into several positions around the root zone, followed by 5 ml of water applied from above to facilitate penetration into the substrate. A nitrogen-free nutrient solution was supplied weekly thereafter.

Noninvasive x-ray microtomography was carried out with an EasyTom 160-150 system (RX Solutions) at the Institute of Applied Mechanics, TU Braunschweig. Full-field scans of intact pots were acquired before inoculation (−4 days) and at 17, 19, 21, and 33 days, as well as 9 and 12 weeks after inoculation (fig. S15). Control plants not subjected to repeated scanning showed no growth differences relative to scanned individuals. Imaging was conducted at 150 kV and 500 μA with 1152 projections over 360°, yielding isotropic voxel sizes of 59 μm. Volumes were reconstructed with X-Act (RX Solutions) using filtered backprojection.

CT datasets (16-bit, 1705 by 1469 by 1469 voxels) were cropped to subvolumes to reduce computational cost, denoised with GPU-accelerated Non-Local Means filtering in Avizo 2024, and edge enhanced using Unsharp Masking in Fiji (*51*–*53*). Similar filtering and segmentation workflows have been successfully applied to complex porous media datasets containing sand, water, and air (*53*–*55*). Pot walls were digitally removed before segmentation, which was performed in Ilastik (*56*). Pixel Classification models were trained on manually labeled datasets and probability maps refined by Object Classification, incorporating intensity- and shape-based descriptors. Classifiers were subsequently applied in batch mode to the full dataset (*56*). This workflow enabled reliable separation of roots, soil, water, and air, although very fine hair roots and boundaries between roots and water remained difficult to resolve due to grayscale similarity and resolution limits (*53*) (fig. S16).

### Sampling of *R. pseudoacacia* tissue

Temporal sampling was at 70 days posttransplantation and 24, 48, and 96 hours and 7, 14, and 21 days postinoculation (dpi), plus 1 year postinoculation (ypi). Entire plants were removed intact, washed, and dissected into leaves, stems, roots, and nodules, immediately frozen in liquid N_2_, and stored at −80°C. For each biological replicate, nodule tissue was harvested from a single individual plant, yielding ~3 g of the fresh nodule material per replicate; nodules were not pooled across plants.

For spatial sampling at 30 dpi, 4-month-old *Robinia* seedlings were carefully removed from pots to minimize root damage and then washed repeatedly in distilled water and blotted dry with cellulose tissue. Nodules with a diameter of ≥0.5 cm located at the mid-root position (defined as 0 cm) were selected and excised. In addition, 2- to 3-cm root segments were collected from positions 5 and 10 cm acropetally relative to the sampled nodules. All nodules and root segments were immediately frozen in liquid N_2_, transported to the laboratory, and stored at −80°C for subsequent transcriptomic analyses.

### Plant RNA extraction and sequencing

Total RNA was extracted from leaf, stem, root, and nodule tissue using TRIzol (Invitrogen, Carlsbad, USA). RNA purity and concentration were determined with a NanoDrop 2000 spectrophotometer (Thermo Fisher Scientific, USA), and integrity was assessed with an Agilent 2100 Bioanalyzer (Agilent Technologies, Santa Clara, CA, USA). Libraries were prepared using the TruSeq Stranded mRNA LT Sample Prep Kit (Illumina, San Diego, CA, USA) and sequenced on the Illumina NovaSeq 6000 platform, generating 150–base pair (bp) paired-end reads. Adapter and low-quality sequences were removed with Trimmomatic v0.39. Clean reads were aligned to the *R. pseudoacacia* reference genome (*14*) (doi: 10.5281/zenodo.14887937) using HISAT2 v2.2.1 (*57*), and gene-level read counts were obtained with HTSeq-count v0.13.5 (*58*). Transcript abundance was quantified as transcripts per million (TPM) in R v4.3.2 from raw counts (*59*). Gene Ontology (GO) (*60*) and Kyoto Encyclopedia of Genes and Genomes (KEGG) (*61*) annotations were assigned.

### DNA extraction, sequencing, and population genomics

Genomic DNA was extracted from young leaves of each plant using a cetyltrimethylammonium bromide (CTAB)–based protocol, followed by RNase A treatment and purification with phenol-chloroform. DNA integrity was assessed by 1% agarose gel electrophoresis, and purity/quantity was determined with a NanoDrop 2000 spectrophotometer (Thermo Fisher Scientific, USA) and Qubit dsDNA BR Assay Kit (Invitrogen, USA).

Genomic DNA libraries were prepared using the TruSeq Nano DNA LT Sample Preparation Kit (Illumina, San Diego, CA, USA). DNA was sheared to ~350-bp fragments on an S220 Focused-ultrasonicator (Covaris, USA), ligated to indexed adapters, PCR amplified, and purified according to the manufacturer’s protocol. Libraries were sequenced on an Illumina HiSeq X Ten platform, producing 150-bp paired-end reads.

Raw reads were quality checked and trimmed with fastp v1.0.1, and clean reads were aligned to the *R. pseudoacacia* reference genome (doi: 10.5281/zenodo.14887937) using BWA-MEM v0.7.19. BAM files were sorted and indexed with SAMtools v1.21 (*62*), and per-base coverage was calculated using samtools depth. Mean depth per BED interval was determined with Bedtools v2.31.1 (*63*). Variant positions within target loci were extracted with samtools mpileup and summarized in tabular format. Coverage profiles and variant distributions were visualized in R (v4.4.0) using ggplot2 and custom scripts.

### Protein extraction, digestion, and LC-MS/MS analysis of nodules

Two to five nodules were disrupted in 300 μl of lysis buffer [10 mM Tris (pH 8) and 1 mM EDTA) using a FastPrep homogenizer with 10 beads of 1 mm in diameter supplemented with 100 μl of 0.1-mm beads (3 × 30 s, 6.5 m s^−1^). Lysates were centrifuged twice for 15 min (12,000*g*, 4°C), and protein concentration in the supernatant was determined using the Roti-Nanoquant assay (Carl Roth, Karlsruhe, Germany). Approximately 50 μg of the protein was denatured in 6 M urea and 1.5 M thiourea, reduced with 0.5 mM dithiothreitol, and alkylated with 5.5 mM iodoacetamide. Digestion was performed in 50 mM ammonium bicarbonate containing 1 mM CaCl_2_ with 1 μg of endoprotease AspN or α-lytic protease for 12 hours and stopped by raising the pH to 11.

Peptides were fractionated using Oasis HLB solid-phase extraction cartridges (1 cm^3^, 10 mg; Waters, Milford, MA, USA). Cartridges were pretreated with 100% acetonitrile and then with 10 mM NH_4_OH in 60% acetonitrile, equilibrated twice with 1 ml of 10 mM NH_4_OH, and loaded with the samples. Flow-through fractions were reapplied to the cartridges, and bound peptides were washed five times with 1 ml of 10 mM NH_4_OH and eluted stepwise with increasing concentrations of acetonitrile to yield eight fractions: (i) 3%, (ii) 6%, (iii) 9%, (iv) 12%, (v) 15%, (vi) 30%, (vii) 45%, and (viii) 80% acetonitrile in 10 mM NH_4_OH. Eluates were collected in low-binding tubes; three-fifths of each volume were dried in a SpeedVac centrifuge (Eppendorf Concentrator Plus). Peptides were desalted using C18 ZipTips (Merck Millipore), eluted with 0.1% formic acid in 60% acetonitrile, and dried again in a SpeedVac.

For liquid chromatography–tandem mass spectrometry (LC-MS/MS), dried peptides were reconstituted in 16 μl of 0.1% formic acid/3% acetonitrile, incubated for 1 hour at room temperature, ultrasonicated for 5 min, and ultracentrifuged at 109,000*g*. Two MS platforms were used:

1) Orbitrap Velos Pro MS (Thermo Fisher Scientific) coupled to a nanoAcquity UPLC system (Waters). Peptides were separated on a BEH C18 column using a 205-min gradient from 3.7 to 99% buffer B (80% acetonitrile and 0.1% formic acid) (*64*). Full MS scans were acquired across mass/charge ratio (*m/z*) 350 to 1750, and singly charged ions were also acquired for collision-induced dissociation (CID) fragmentation in MS^2^ scans. Data were processed using MaxQuant with label-free quantification (LFQ) enabled.

2) Orbitrap Fusion MS (Thermo Fisher Scientific) coupled to a Dionex Ultimate 3000 UPLC system. Peptides were separated on a 200-cm μPAC RP C18 column (PharmaFluidics) using a 210-min gradient at 300 nl min^−1^ with buffer A [0.1% formic acid and 5% dimethyl sulfoxide (DMSO)] and buffer B (80% acetonitrile, 0.1% formic acid, and 5% DMSO): 0 to 7 min, 3.7% B; 7 to 125 min, 3.7 to 31.3% B; 125 to 165 min, 31.3 to 62.5% B; 165 to 172 min, 62.5 to 90% B; 172 to 177 min, 90% B; 177 to 182 min, 90 to 3.7% B; and 182 to 210 min, 3.7% B. The instrument, controlled with the Xcalibur software, was operated in “top speed” mode with higher energy collisional dissociation (HCD) fragmentation. Full MS scans were acquired across *m/z* 350 to 1700 in the Orbitrap at 120,000 resolution (full width at half maximum at *m/z* 400) with a lock mass of 445.12003. Precursor ions [±10 parts per million (ppm)] were isolated in the quadrupole (1.6 *m/z* window), fragmented in the ion trap, and analyzed with dynamic exclusion set to 13 s.

### Proteomics database analysis

Analyses of MS and MS/MS data were performed using MaxQuant (v1.6.17.0; Max Planck Institute of Biochemistry, Martinsried, Germany; www.maxquant.org). Searches were conducted against a protein database derived from the *R. pseudoacacia* genome with the following parameters: enzyme specificity set to AspN/α-lytic protease with up to five missed cleavages (for AspN) or six missed cleavages (for α-lytic protease) allowed; precursor mass tolerance of 5 ppm; fragment mass tolerance of 0.6 Da; variable modifications included methionine oxidation and protein N-terminal acetylation; carbamidomethylation of cysteine was set as a fixed modification. The FDR was controlled at 1% on the peptide and protein levels. Only proteins with at least one unique peptide identified in two or more MS/MS scans were retained.

### Cloning of NPG constructs for recombinant expression

Genomic DNA and total RNA were extracted from *R. pseudoacacia* nodules using a CTAB-based protocol. Briefly, frozen nodules were ground to a fine powder in liquid nitrogen, suspended in prewarmed CTAB extraction buffer [2% CTAB, 100 mM tris-HCl (pH 8.0), 20 mM EDTA, 1.4 M NaCl, and 1% polyvinylpyrrolidone], and incubated at 65°C for 30 min. After extraction with chloroform:isoamyl alcohol (24:1), the aqueous phase was recovered, and nucleic acids were precipitated with isopropanol, washed in 70% ethanol, and dissolved in nuclease-free water. DNA was treated with RNase A and RNA with DNase I to remove residual contaminants before downstream analyses. RNA was reverse transcribed to cDNA using the ProtoScript II Reverse Transcriptase (New England Biolabs, USA).

For bacterial expression, the CDSs of mature NPG1 and NPG2 (lacking the predicted N-terminal signal peptide) were amplified by PCR using gene-specific primers (table S6) and cloned into a custom isopropyl-β-d-thiogalactopyranoside (IPTG)–inducible expression vector derived from pET-19b (Novagen, Merck Millipore, Darmstadt, Germany). The vector was generated by inserting a TrxA fragment fused to a tobacco etch virus (TEV) protease cleavage site into pET-19b, using the pET Trx His_6_ LIC cloning vector (2Tc-T; Addgene plasmid #37239) as a template. Assembly was performed with the NEBuilder HiFi DNA Assembly (New England Biolabs, USA), ensuring in-frame fusion with the TEV site to yield peptides without additional residues after cleavage. For the Strep-tagged construct, a Strep-Tag II sequence was introduced directly after the TEV site by site-directed mutagenesis (Q5 Site-Directed Mutagenesis Kit, New England Biolabs, USA), producing a TEV-cleavable, Strep-Tag II-NPG2 fusion. Constructs were transformed into *Escherichia coli* BL21(DE3) cells (Thermo Fisher Scientific, USA) and grown at 37°C in LB medium to OD_600_ ≈ 0.5 before induction with 1 mM IPTG, followed by incubation at 22°C for 20 hours. Cells were harvested from 1-liter cultures by centrifugation (10,000*g*, 20 min, 4°C), washed in Buffer W [100 mM Tris (pH 8.0), 150 mM NaCl, and 1 mM EDTA], transferred to 50-ml tubes, and centrifuged again (10,000*g*, 20 min, 4°C). The pellets were then flash frozen in liquid N_2_. Lysis was performed enzymatically with lysozyme (1 mg ml^−1^) followed by sonication (50% amplitude, six cycles of 15 pulses, and 30-s cooling intervals on ice) using a SONOPULS HD 60 ultrasonic homogenizer (Bandelin, Berlin, Germany). The lysate was clarified by centrifugation (50,000*g*, 30 min, 4°C) and filtered (0.2 μm). Recombinant proteins were purified on an ÄKTA Purifier system (Cytiva, Marlborough, USA) using Protino Ni-NTA columns (Macherey-Nagel, Düren, Germany) according to the manufacturer’s instructions and buffer exchanged to PBS using PD-10 desalting columns (Cytiva, Marlborough, USA). For NPG2 requiring tag removal, His-tagged protein was digested overnight at 4°C with TEV protease (New England Biolabs, USA) at a 1:20 (w/w) enzyme-to-substrate ratio. Cleaved samples were further purified on the ÄKTA system using a Strep-Tactin XT column (IBA Lifesciences, Göttingen, Germany) to separate NPG2 peptides from TEV protease and TrxA, following the manufacturer’s protocol. Purified proteins were used for *M. robiniae* exposure assays and for rabbit polyclonal antibody generation.

For fluorescence localization studies, genomic fragments spanning exon 1, intron, and exon 2 of NPG1 and NPG6 were amplified from *R. pseudoacacia* genomic DNA using gene-specific primers (table S6). These fragments were cloned into the Gateway-compatible binary vector pDest-GW-Venus (*65*) downstream of the native promoter using the NEBuilder HiFi DNA Assembly (New England Biolabs, USA), resulting in NPG1::Venus and NPG6::Venus translational fusions. The constructs were sequence verified and subsequently used for Agrobacterium-mediated plant transformation.

### Agrobacterium-mediated plant transformation and transient expression assays

Agrobacteria were transformed with the binary constructs using electrocompetent cells and plated on selective agar. After 2 days at 28°C, single colonies were picked and cultured overnight in selective liquid medium. The resulting cultures were diluted into fresh medium and grown to an OD_600_ of ~0.8 to 1.0 and then pelleted and resuspended in infiltration buffer [10 mM MgCl_2_, 10 mM MES (pH 5.6), and 100 to 200 μM acetosyringone]. Cell suspensions were adjusted to a final OD_600_ of 0.3 to 0.4 and mixed with the viral silencing suppressor strain (19K). After a 2- to 3-hour induction period at room temperature in the dark, the suspensions were infiltrated into fully expanded leaves of 4- to 6-week-old *N. benthamiana* plants by gentle pressure using a needleless syringe applied to the abaxial side of the leaf. Plants were returned to the growth chamber and incubated for 48 to 72 hours before imaging of Venus fluorescence by confocal microscopy.

### Purification of NPG-specific antibodies

Rabbit polyclonal serum (5 ml) generated against NPG1 (Eurofins Genomics, Germany; 28-day Speedy program) was purified on a Protein A column (Cytiva, Marlborough, USA) following the manufacturer’s instructions. The eluate was subsequently affinity purified using Strep-tagged NPG2 immobilized on a Strep-Tactin XT column (IBA Lifesciences, Göttingen, Germany). Bound antibodies were eluted with 0.1 M citric acid (pH 3.0) directly in 500-μl fractions into Eppendorf tubes preloaded with 200 μl of 1 M tris-HCl (pH 9.0) to achieve immediate neutralization. The eluate was buffer exchanged into PBS using PD-10 columns (Cytiva, Marlborough, USA) and adjusted to 1 mg ml^−1^. Purified antibodies were flash frozen in liquid nitrogen and stored at −80°C until use.

### Nodule section preparation and immunostaining

Freshly harvested *R. pseudoacacia* nodules were fixed in 4% (w/v) paraformaldehyde (PFA; Sigma-Aldrich, St. Louis, USA) in PBS (Gibco, Thermo Fisher Scientific, Waltham, USA) for 8 hours at room temperature or overnight at 4°C, rinsed three times in PBS, and cryoprotected in a sucrose gradient (10% for 1 hour, 20% for 1 hour, 30% for 1 hour, and 40% at least overnight until sectioning at 4°C; Sigma-Aldrich). Samples were embedded in OCT compound (Tissue-Tek, Sakura Finetek, Tokyo, Japan) and sectioned at −20°C to a thickness of 20 μm using a cryotome (CM1950, Leica Microsystems, Wetzlar, Germany). Sections were air dried at room temperature, postfixed in 4% PFA in PBS for 10 min, washed three times in PBS, and blocked for 1 hour at room temperature in blocking buffer [0.25% Triton X-100 (Sigma-Aldrich) and 5% horse serum (Thermo Fisher Scientific) in PBS]. Sections were incubated overnight at 4°C with affinity-purified anti-NPG antibody (1:200; custom-generated, Eurofins Genomics, Ebersberg, Germany) diluted in antibody buffer (0.1% Triton X-100 and 5% horse serum in PBS). After three 10-min washes in TBST [tris-buffered saline with 0.1% Tween 20 (Sigma-Aldrich)], sections were incubated for 2 hours at room temperature with Alexa Fluor 488–conjugated goat anti-rabbit secondary antibodies (Invitrogen, Thermo Fisher Scientific; A11034 or A11032; 1:500) and/or with DAPI (Sigma-Aldrich; D9564; 1:500). Sections were washed three times in TBST for 10 min each and mounted in ProLong Diamond Antifade Mountant (Invitrogen, Thermo Fisher Scientific). Samples were imaged within 48 hours of staining.

### Confocal microscopy

Confocal imaging was performed using a Zeiss LSM 880 system (Carl Zeiss, Oberkochen, Germany) equipped with an Axio Observer Z1 inverted stand. For *N. benthamiana* transient expression, leaves were imaged 4 days after *Agrobacterium tumefaciens* infiltration with NPG-YFP constructs. YFP was excited at 488 nm, and emission was collected at 505 to 550 nm. For *R. pseudoacacia* nodule sections prepared as described above, Alexa Fluor 488 was excited at 488 nm and emission was collected at 505 to 550 nm; DAPI was excited at 364 nm, and emission was collected at 385 to 490 nm. Images were acquired using either a Plan-Apochromat 20×/0.8 M27, a C-Apochromat 40×/1.2 water-immersion objective (Carl Zeiss) or an EC Plan-Neofluar 100×/1.3 oil-immersion objective (Carl Zeiss). Sequential scanning was applied to avoid channel cross-talk. Bright-field images were captured using transmitted-light detection. All images were processed in Fiji v1.53 (NIH, Bethesda, USA) for background subtraction and contrast adjustment; identical detector settings and processing parameters were applied to compared images. Line plot analysis of NPG immunofluorescence was performed in Fiji (Plot Profile function) by extracting normalized gray values along the base-apex axis of the nodule (root-attached base to the apical meristem); profiles from multiple sections were averaged across ≥3 biological replicates.

### Peptide synthesis

Five synthetic peptides corresponding to NPG-derived sequences (table S5) were synthesized at >95% purity by Nanjing TY Peptide Co. (Nanjing, China) and verified by reversed-phase high-performance liquid chromatography (HPLC) and electrospray ionization MS. Lyophilized peptides were stored at −20°C under desiccation until use.

### LC-MS analysis

Liquid chromatography–high-resolution mass spectrometry (LC-HRMS) was performed on an Ultimate 3000 UHPLC system (Thermo Fisher Scientific, Bremen, Germany) coupled to a Q Exactive Orbitrap high-performance benchtop mass spectrometer (Thermo Fisher Scientific) operating in positive electrospray ionization mode. Separation was achieved on an Accucore C18 column (2.1 mm by 100 mm, 2.6-μm particle size; Thermo Fisher Scientific). Gradient elution with mobile phases consisting of acetonitrile containing 0.1% (v/v) formic acid and H_2_O containing 0.1% (v/v) formic acid was used. The gradient started at 5:95 (acetonitrile:H_2_O), changed to 2:98 over 10 min, and was held at 2:98 for an additional 4 min. The flow rate was set to 0.2 ml/min, and the injection volume was 3 μl. Mass spectra were acquired across an *m/z* range of 130 to 3000.

### NMR analysis of peptides

All 1D (^1^H) and 2D [^1^H-^1^H COSY, TOCSY (total correlation spectroscopy), HSQC (heteronuclear single-quantum coherence), NOESY, and rotating-frame Overhauser enhancement spectroscopy (ROESY)] NMR spectra were acquired on a Bruker Avance III 500 MHz spectrometer equipped with a cryoprobe at 298 K. Samples (0.5 to 1 mM) were dissolved in 5:1 10 mM sodium phosphate buffer (pH 7.4) and DMSO-*d*_6_. Data were processed with TopSpin (v3.6, Bruker). Chemical shifts are reported in parts per million (ppm) relative to the residual solvent signals: δ(DMSO-*d*_6_) = 2.50 ppm for ^1^H and δ(DMSO-*d*_6_) = 39.52 ppm for ^13^C.

### CD spectroscopy

CD spectra were recorded on a J-815 spectropolarimeter (JASCO, Tokyo, Japan) at 20°C using high-precision quartz cuvettes (1 mm in path length; Hellma Analytics). Protein and peptide samples were prepared at 50 μM in 10 mM sodium phosphate buffer (pH 7.4) and filtered before use. Spectra were acquired from 185 to 280 nm with a 1-nm bandwidth, 0.5-nm step size, and 1-s averaging time per point. Three scans per sample were averaged and expressed as mean residue ellipticity (mdeg) relative to the buffer baseline. Data acquisition and processing were carried out using the JASCO J-800 control software (v. 2.08.04).

### Recombinant peptide exposure assay

*M. robiniae* DSM 100022 was cultured in yeast extract mannitol (YEM) broth [yeast extract (0.4 g liter^−1^), mannitol (10 g liter^−1^), NaCl (0.1 g liter^−1^), MgSO_4_·7H_2_O (0.2 g liter^−1^), and K_2_HPO_4_ (0.2 g liter^−1^) (pH 6.8 to 7.0)] at 28°C with shaking (180 rpm) until mid-logarithmic phase (OD_600_ = 0.4 to 0.6). Cells were harvested by centrifugation (4000*g*, 10 min, 4°C), washed twice with PBS, and resuspended in fresh YEM broth to OD_600_ = 0.2. Equal volumes (500 μl each) of the cell suspension and peptide solution (digested or undigested recombinant NPG2; 0 to 250 μM final concentration) were mixed in 1.5-ml microcentrifuge tubes, resulting in a starting OD_600_ = 0.1. Cultures were incubated for 2 hours at 28°C with gentle agitation. Control samples without peptide were processed in parallel. After incubation, cultures were serially diluted in PBS and 100-μl aliquots were spread onto YEM agar plates. Plates were incubated at 28°C for 3 to 5 days, and bacterial growth area was quantified from scanned plate images using Fiji v1.53 (NIH, Bethesda, USA) by threshold-based segmentation. For RNA-seq, after the 2-hour incubation, 50% (v/v) RNAlater was added to an additional aliquot, which was then flash frozen in liquid nitrogen. The entire experiment was performed independently three times from culture initiation, and results were averaged for fig. S8.

### qPCR analysis of NPG2-responsive *M. robiniae* genes

For reverse transcription qPCR validation, *M. robiniae* cultures were treated with recombinant NPG peptides using the same exposure conditions described for the recombinant peptide exposure assay. In addition to NPG2, two further peptides (NPG-IV and NPG-V; table S5) were tested in parallel. After 2-hour incubation, samples were centrifuged and 1 ml of RNAprotect Bacteria Reagent (QIAGEN, Hilden, Germany) was added and stored at room temperature for 30 min before freezing at −80°C. Pellets were thawed on ice and centrifuged (8000*g*, 10 min, 4°C), and subsequently the supernatant was removed. Pellets were resuspended in TE buffer containing lysozyme (15 mg ml^−1^) and Proteinase K (20 μl per sample), following the enzymatic lysis step of the RNeasy Protect Bacteria Mini Kit (QIAGEN, Hilden, Germany). After 10-min incubation, total RNA was purified according to the manufacturer’s instructions, including the on-column genomic DNA digestion step as described.

RNA concentration was determined using a Qubit fluorometer (Thermo Fisher Scientific, Waltham, USA). Residual genomic DNA was removed by in-solution digestion with TURBO DNase (Invitrogen, Carlsbad, USA) according to the manufacturer’s protocol, and the absence of DNA contamination was confirmed by no reverse transcriptase (−RT) controls. First-strand cDNA was synthesized from 200 ng of total RNA using the LunaScript RT SuperMix (New England Biolabs, Ipswich, USA) in 20-μl reactions following the manufacturer’s standard protocol. One microliter of the resulting cDNA was used as a template for qPCR. qPCR was performed using the Luna Universal qPCR Master Mix (2×) (New England Biolabs, Ipswich, USA) on a LightCycler 96 (Roche) under the manufacturer’s recommended cycling conditions. Each reaction was run in two technical replicates; replicate pairs differing by more than 1 Cq were excluded from analysis. Cq values were obtained, and ΔCq values were calculated using the LightCycler 96 Software (version 1.1, Roche) with *rpsB* and/or *rpsU* used for normalization and PBS-treated samples serving as the reference condition. Target genes analyzed were *nifH*, *nirB*, and *mbfA*. Primer sequences are listed in table S7. Primer efficiencies were determined from standard curves generated from serial dilutions of template (0.5 to 50 ng input equivalent) and calculated from the slope of the regression [*E* = 10^(−1/slope)^]. All primer efficiencies ranged between 1.8 and 2.2 with *R*^2^ ≥ 0.98 (table S7). No template and −RT controls were included in all runs.

### Prokaryotic RNA isolation, library preparation, and bioinformatics analysis

Total RNA was extracted from *M. robiniae* cultures using the RNAprep Pure Cell/Bacteria Kit (TIANGEN, Beijing, China; catalog no. DP430) according to the manufacturer’s protocol. RNA purity and concentration were measured with a NanoDrop 2000 spectrophotometer (Thermo Scientific, Waltham, USA), and RNA integrity was assessed using an Agilent 2100 Bioanalyzer (Agilent Technologies, Santa Clara, USA). RNA integrity numbers (RINs) ranged from 5.9 to 10.0, with all samples showing clear 23*S*/16*S* ribosomal RNA (rRNA) peaks; for bacterial RNA, RIN values ≥ 5.9 were deemed acceptable for sequencing as modest variation in integrity has minimal impact on prokaryotic transcriptome profiling. Samples meeting these criteria for purity, quantity, and integrity were used for downstream library construction. rRNA was removed using the TIANSeq rRNA Depletion Kit (TIANGEN, Beijing, China), and strand-specific libraries were prepared with the VAHTS Universal V6 RNA-seq Library Prep Kit (Vazyme, Nanjing, China) following the manufacturer’s instructions.

RNA-seq was performed on an Illumina platform by OE Biotech Co. Ltd. (Shanghai, China). Bioinformatics analysis was carried out using the company’s standard prokaryotic RNA-seq pipeline. In brief, raw reads in FASTQ format were processed with Trimmomatic (*66*) to remove adapter sequences, low-quality bases, and reads containing ambiguous nucleotides. The resulting clean reads were aligned to the *M. robiniae* DSM 100022 reference genome (GCF_040545535.1) using Rockhopper2 (*67*). Gene expression levels were quantified in fragments per kilobase per million mapped reads (FPKM).

Differential expression analysis between treatment groups was performed using DESeq2 (estimateSizeFactors and nbinomTest functions), applying thresholds of |fold change| ≥ 1.5 and *q* value < 0.05. Functional enrichment analysis of differentially expressed genes (DEGs) was conducted using the hypergeometric test for GO terms and KEGG pathways. For KEGG, the top 20 enriched pathways with PopHits ≥ 5 were ranked by −log_10_(*P* value), and pathway-gene relationships were visualized with chord diagrams.

GSEA was performed with the R package fgsea on ranked expression datasets using GO, KEGG, and MSigDB gene sets. Additional analyses included gene prediction, operon structure prediction, Shine-Dalgarno sequence detection, and SNP/INDEL calling using samtools and BCFtools .

### Structural modeling

Full-length peptide sequences of all seven NPG isoforms were submitted as queries to the AlphaFold3 server (https://alphafoldserver.com/, Google DeepMind, London, UK) for structural prediction. Models were generated using default parameters, and the highest-ranked model for each sequence, based on the pLDDT score, was selected for downstream analysis.

### Phylogenetic tree generation

Protein sequences of the NPG family were aligned using MAFFT v7.526 with the L-INS-i strategy, an iterative refinement method incorporating local pairwise alignment, to account for conserved motifs and minimize the introduction of gaps. The resulting multiple sequence alignment was used to construct a maximum likelihood phylogenetic tree in IQ-TREE v2.3.6 (*68*). The best-fit substitution model was determined by ModelFinder, which selected the JTT model based on Bayesian Information Criterion (BIC) scores. Branch support was assessed with 1000 ultrafast bootstrap replicates to ensure confidence in the inferred topology. The final unrooted tree was visualized in R using the ggtree (v3.12) package, with bootstrap values annotated to highlight the reliability of individual branches.

The recently published papilionoid phylogeny was obtained in Newick format and visualized with the R package ggtree. Species presence in Hologalegina (IRLC and Robinioids) was annotated manually. Growth form (tree versus shrub versus herbaceous) was determined manually from published floras and taxonomic descriptions. Genome assemblies of available Hologalegina taxa were retrieved from the NCBI Genome portal (accessed August 2025). The presence or absence of NPG or NCR loci was assessed by BLAST+ (v2.15.0) (*69*) searches against the corresponding genome assemblies (GCF/GCA accessions). No significant hits were detected for NPG peptides in any sequenced Robinioid genome. Similarly, representative NCR peptides (*M. truncatula* NCR001, NCR169, and NCR211) produced no hits outside IRLC genomes. Results were manually cross-checked with the respective NCBI genome annotations and mapped onto the phylogeny.

### Homology search across Papilionoideae genomes

To assess the broader distribution of NPG homologs within legumes, an initial tBLASTn search was conducted using the NCBI web interface across all publicly available genome assemblies of over 400 Fabaceae species. This preliminary screen did not yield any significant hits outside the Papilionoideae subfamily, suggesting that NPG genes may be restricted to this lineage or are highly diverged in others.

On the basis of this observation, a more detailed homology search was performed across Papilionoideae species. Genome assemblies were downloaded from the NCBI (https://ncbi.nlm.nih.gov) according to the following criteria: Among all available assemblies from Papilionoideae species, one representative per species was selected if published between 1 January 2022 and 8 April 2025. When multiple assemblies existed for a given species, preference was given to RefSeq assemblies (GCF) over GenBank assemblies (GCA), followed by the highest assembly level and the scaffold N50. In total, genome assemblies from 105 species were included.

Both tBLASTn and BLASTn searches were conducted using the BLAST+ suite (v. 2.16.0+) (*69*). Custom BLAST databases were created for each genome using makeblastdb. Peptide sequences from NPG1 to NPG6/7 were used as queries for tBLASTn, whereas CDSs and individual exon sequences were used for BLASTn analyses. All searches were conducted with default parameters, using the -task BLASTn flag for nucleotide-based searches.

To eliminate redundancy, BLASTn results were filtered such that, if both a full CDS hit and an exon-specific hit occurred at the same locus, only the CDS hit was retained. Query sequences were translated in the correct reading frame, whereas alignment sequences were translated in all six possible frames for comparison.

### Statistics and reproducibility

Unless otherwise stated, all experiments were performed with at least three independent biological replicates, each consisting of material derived from separate plants grown under identical conditions. Technical replicates were included where indicated. Data are presented as means ± SD unless noted. For quantitative comparisons between two groups, statistical significance was assessed using two-tailed Student’s *t* tests after confirming normality (Shapiro-Wilk test) and homogeneity of variances (Levene’s test). Linear regression was performed for transcriptional expression gradients along the root axis, and the coefficient of determination (*R*^2^) is reported. PCA was conducted in R v4.3.2 using the prcomp function with centered and scaled variables. Differential gene expression in RNA-seq datasets was analyzed using DESeq2 (*70*) v1.40.2 with Benjamini-Hochberg FDR correction; genes with adjusted *P* < 0.05 and |log_2_ fold change| > 1 were considered significant. GO and KEGG enrichments were tested using a hypergeometric test with FDR correction. All statistical analyses were performed in R v4.3.2 or GraphPad Prism v10.0. Experiments were repeated independently with similar results, and representative data are shown.

## Supplementary Materials

### The PDF file includes:

Figs. S1 to S16 Tables S1 to S7 Legends for movies S1 to S4 References
