# Supplementary material for: Symbiotic peptides modulate rhizobial physiology without terminal differentiation
Source: Sci Adv. 2026 Jun 26;12(26):eaed2816. doi: 10.1126/sciadv.aed2816 (PMC13308600; doi:10.1126/sciadv.aed2816)
Supplement: Supplementary file 1 — Figs. S1 to S16 Tables S1 to S7 Legends for movies S1 to S4 References [file sciadv.aed2816_sm.pdf]

Science Advances
AAAS

Supplementary Materials for

Symbiotic peptides modulate rhizobial physiology without terminal differentiation

Bin Hu *et al.*

Corresponding author: Kevin D. Oliphant, kevin.oliphant@bioch.ox.ac.uk

Sci. Adv. **12**, eaed2816 (2026)
DOI: 10.1126/sciadv.aed2816

The PDF file includes:

Figs. S1 to S16
Tables S1 to S7
Legends for movies S1 to S4
References

Other Supplementary Material for this manuscript includes the following:

Movies S1 to S4

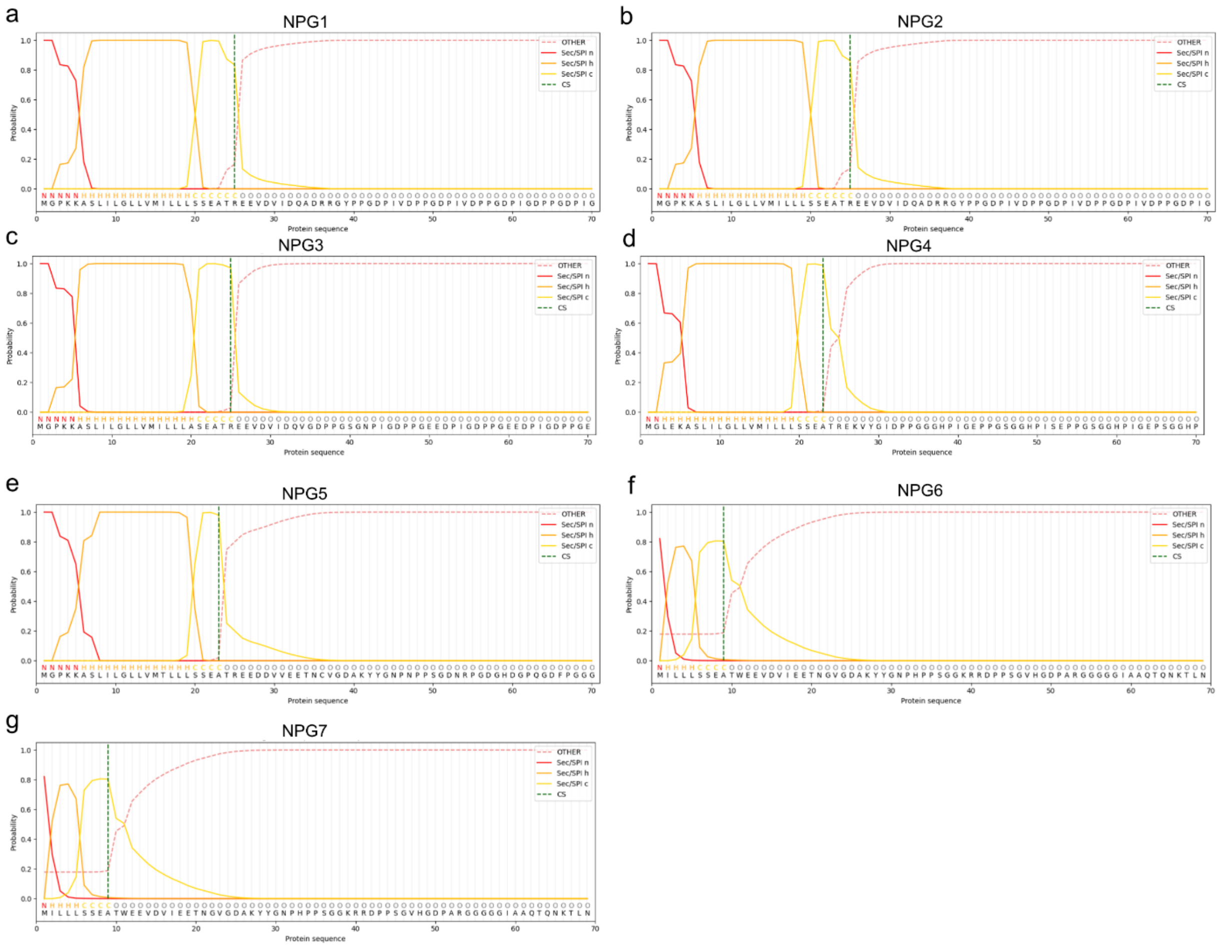

Fig. S1. SignalP 6.0 predictions for NPG proteins. Signal peptide prediction was performed using SignalP 6.0 (*71*) for NPG1-NPG7 **(A-G)**. For each protein, the probability scores for signal peptide, transmembrane region and other sequence features are plotted along the N-terminal region, with the predicted cleavage site indicated.

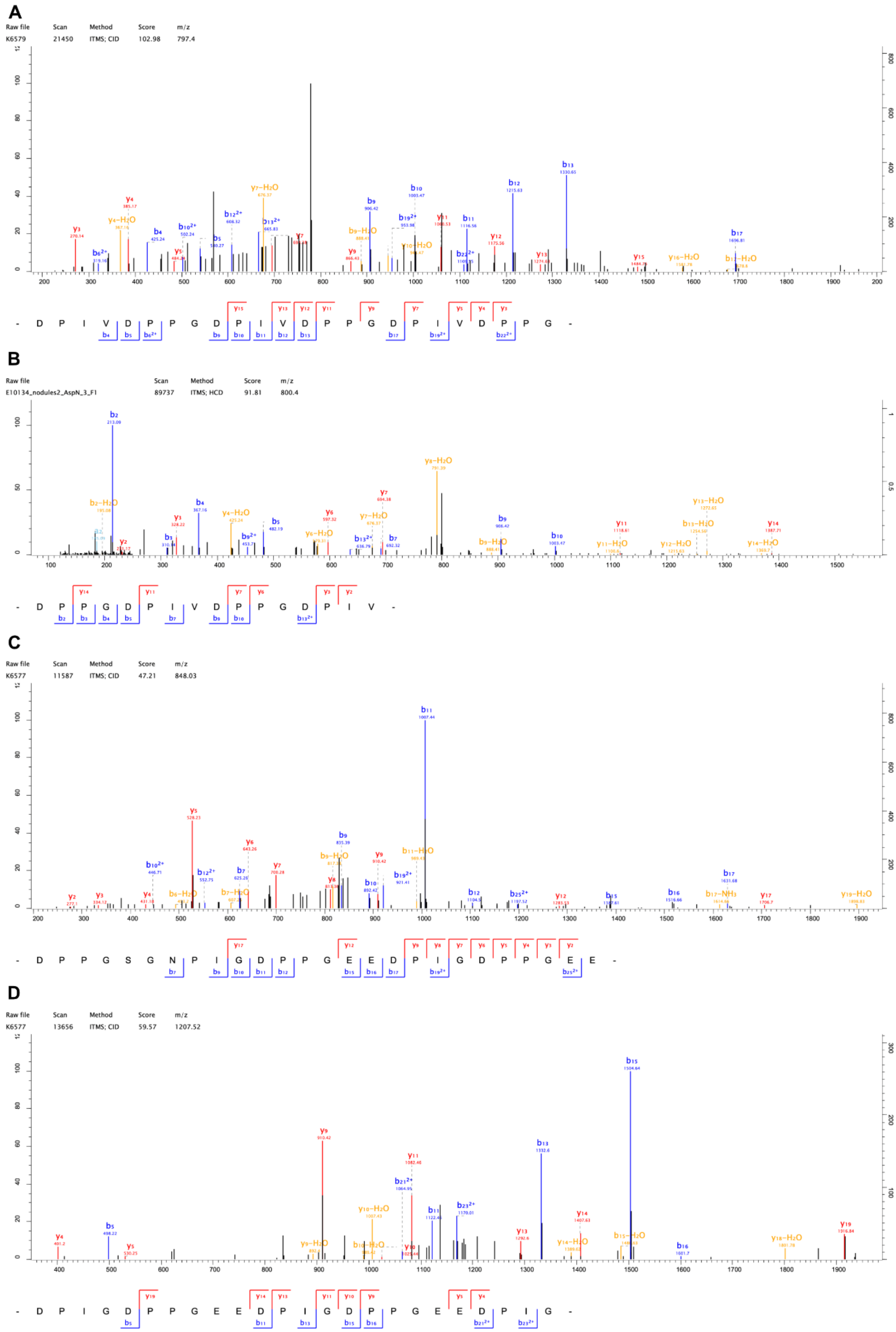

Figure S2. Annotated MS2 spectra of the unique peptides identified for NPG2 and NPG3. (**A**) The peptide DPIVDPPGDPIVDPPGDPIVDPPG, which originates from an AspN

digest of NPG2, was fragmented by collision induced dissociation (CID). (**B**) Peptide DPPGDPIVDPPGDPIV originating from an AspN digest of NPG2 and fragmented by higher-energy collisional dissociation (HCD). (**C**) Peptide DPPGSGNPIGDPPGEEDPIGDPPGEE originating from an AspN digest of NPG3 and fragmented by CID. (**D**) Peptide DPIGDPPGEEDPIGDPPGEEDPIG originating from an AspN digest of NPG3 and fragmented by CID.

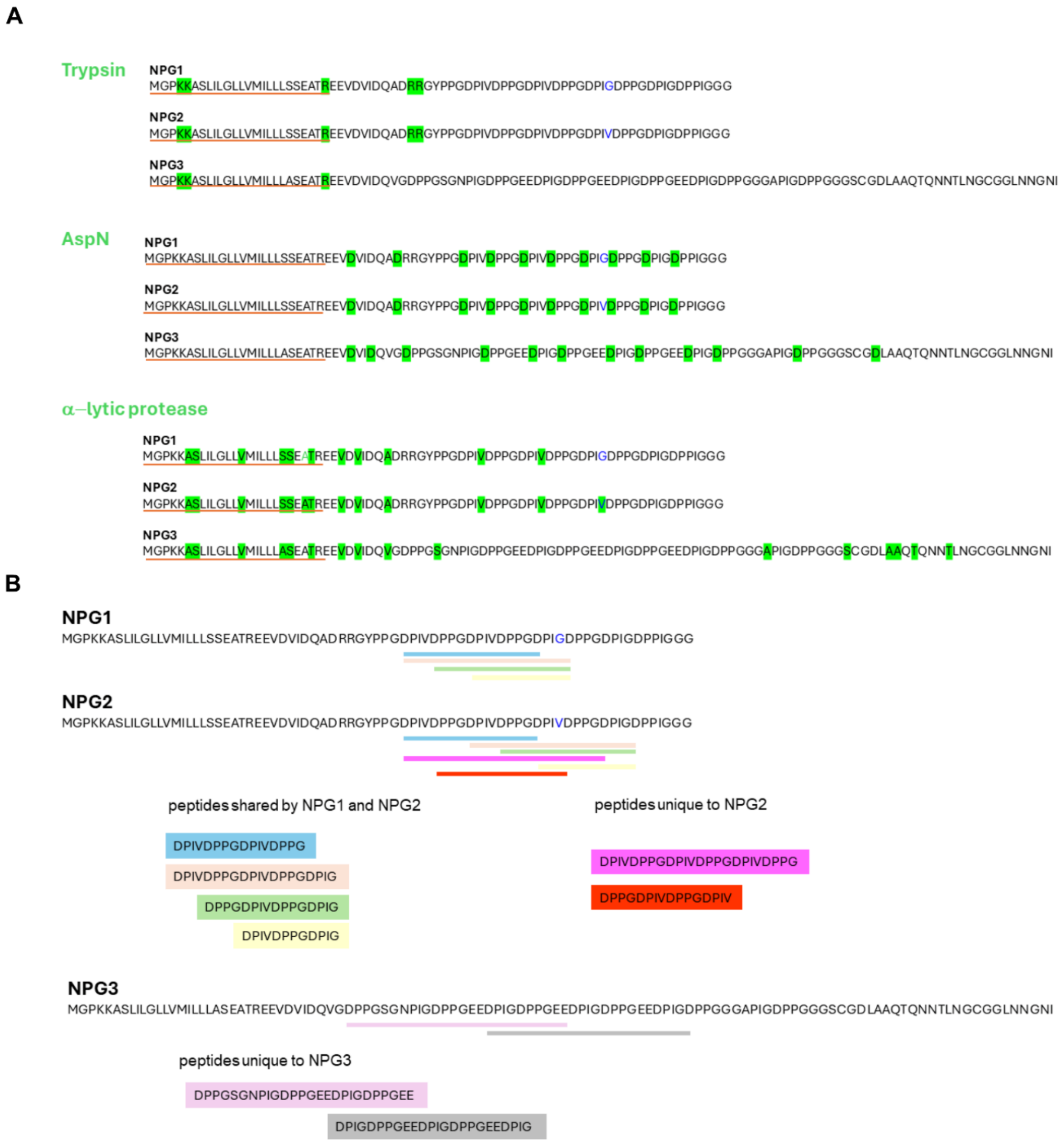

Fig. S3. Theoretical digestion of proteins NPG1, NPG2 and NPG3 and the corresponding peptides that were detected experimentally by MS. (**A**) Theoretical digestion of proteins NPG1, NPG2 and NPG3 using the proteases trypsin, AspN and α-lytic protease. The amino acids recognized by the respective proteases are marked in green. (**B**) Peptide sequences of the unique peptides identified by MS for proteins NPG1, NPG2 and NPG3, and mapping of the peptides to the corresponding protein sequences.

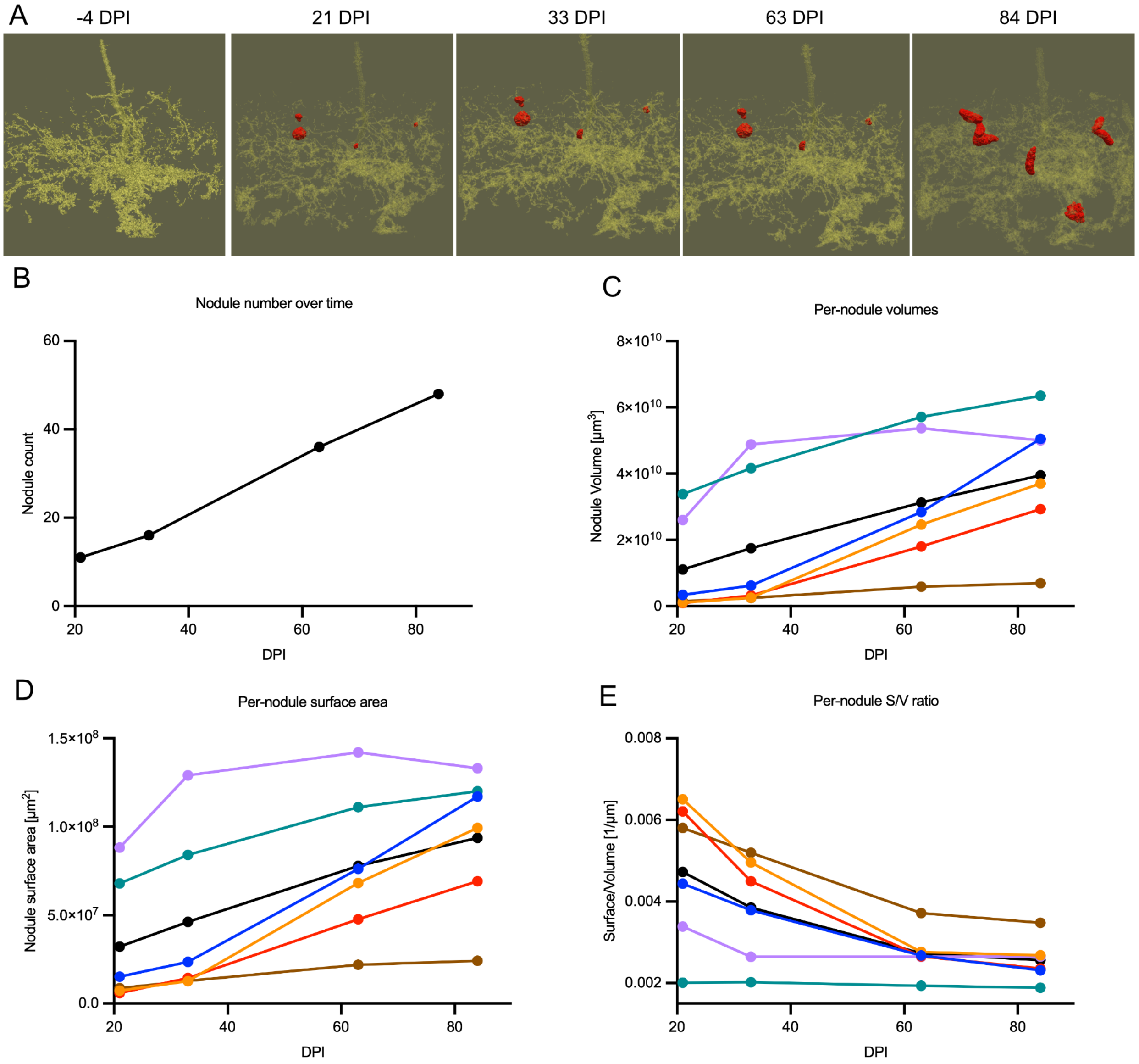

Fig. S4. Quantitative Computed Tomography (CT) monitoring of nodule development in *R. pseudoacacia.* **(A)** 3D CT reconstructions of root systems before inoculation (-4 d) and at ~17, 19, 33, and 89 days post-inoculation. Roots are shown in yellow and nodules in red. **(B)** Nodule number, **(C)** per-nodule volumes, **(D)** per-nodule surface areas, and **(E)** per-nodule surface-to-volume ratios quantified from sequential CT datasets. Colored lines correspond to six individual nodules, consistently assigned across panels C-E; the black line indicates the mean trajectory.

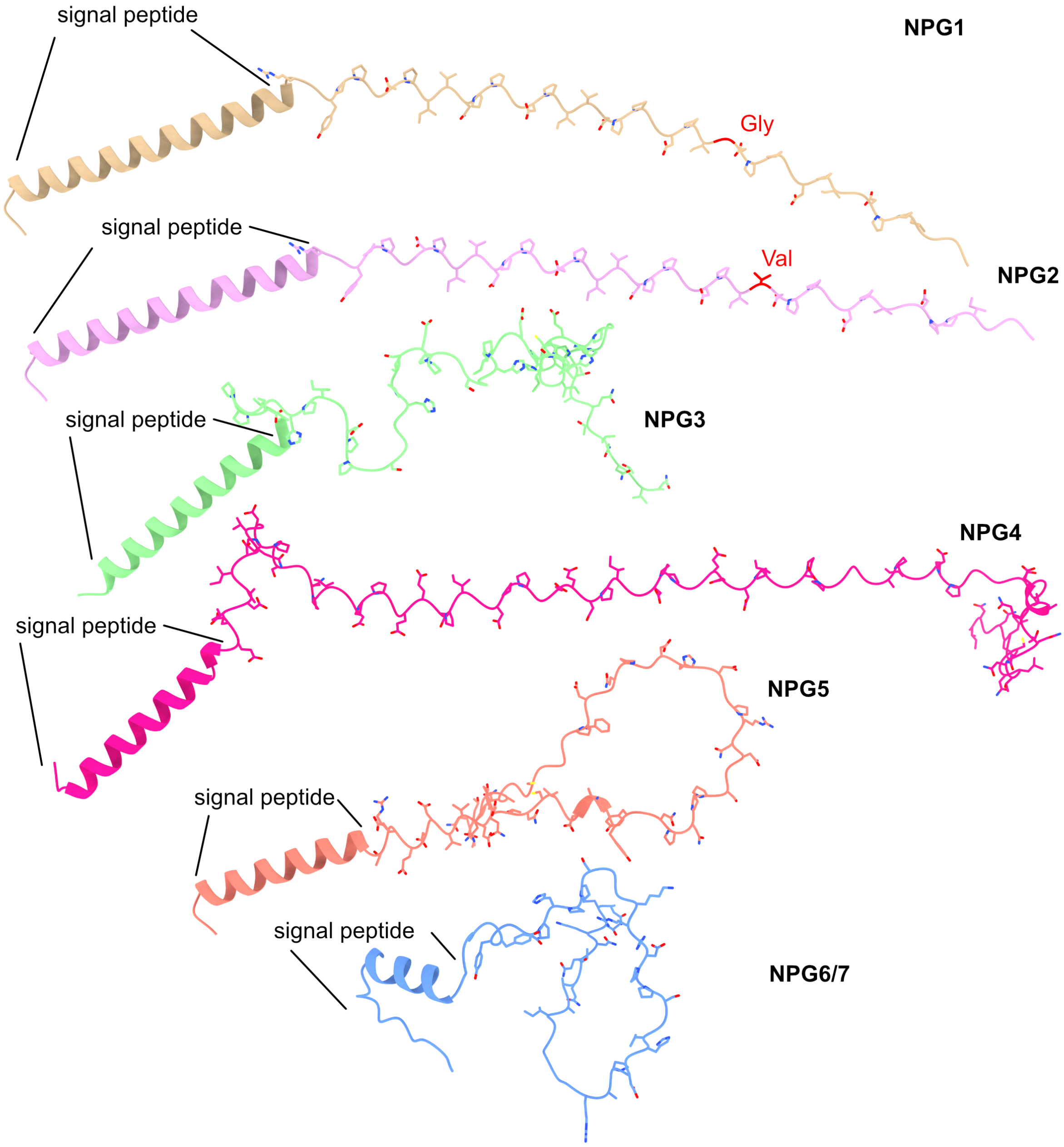

Fig. S5. AlphaFold 3 models of NPG proteins. Predicted three-dimensional structures of NPG1-NPG7 generated with AlphaFold 3 (*29*). Models are shown as ribbon representations of the full-length sequences, including the predicted N-terminal signal peptides.

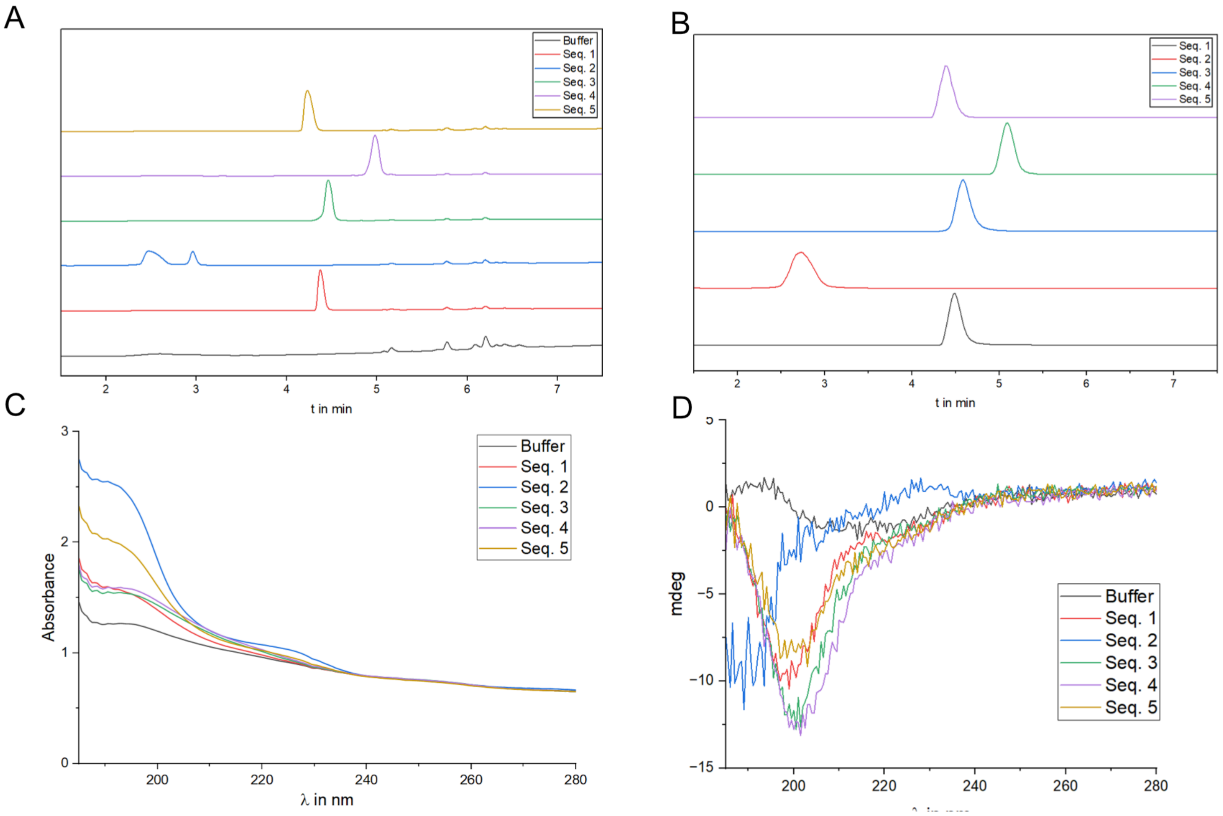

Fig. S6. Biophysical and biochemical analyses of synthetic NPG peptide modules. (A) HPLC-PDA chromatograms showing the UV absorption profiles of peptide samples within a retention time range of 1.5-7.5 min. No evidence of complex formation was observed, indicating that the peptides remain in isolated form in solution. **(B)** HPLC-EIC chromatograms of peptide sequences 1-5 recorded within a retention time range of 1.5-7.5 min. Signals correspond to the $[M + H + MeCN]^+$ adduct ions with the following *m/z* values: Seq. 1 = 987.4629, Seq. 2 = 778.3955, Seq. 3 = 850.4305, Seq. 4 = 1640.8166, and Seq. 5 = 1705.8140. **(C)** UV absorbance spectra of peptide solutions and phosphate buffer recorded between 185-280 nm using a J-815 CD spectrometer at 20 °C in 1 mm pathlength cuvettes. The phosphate buffer shows minimal absorbance, whereas elevated absorbance in peptide-containing samples reflects characteristic peptide bond transitions. **(D)** Circular dichroism (CD) spectra of peptide solutions and phosphate buffer recorded between 185-280 nm using a J-815 CD spectrometer at 20 °C in 1 mm pathlength cuvettes. All peptide sequences exhibit spectra characteristic of disordered peptides, with no evidence of defined secondary structure.

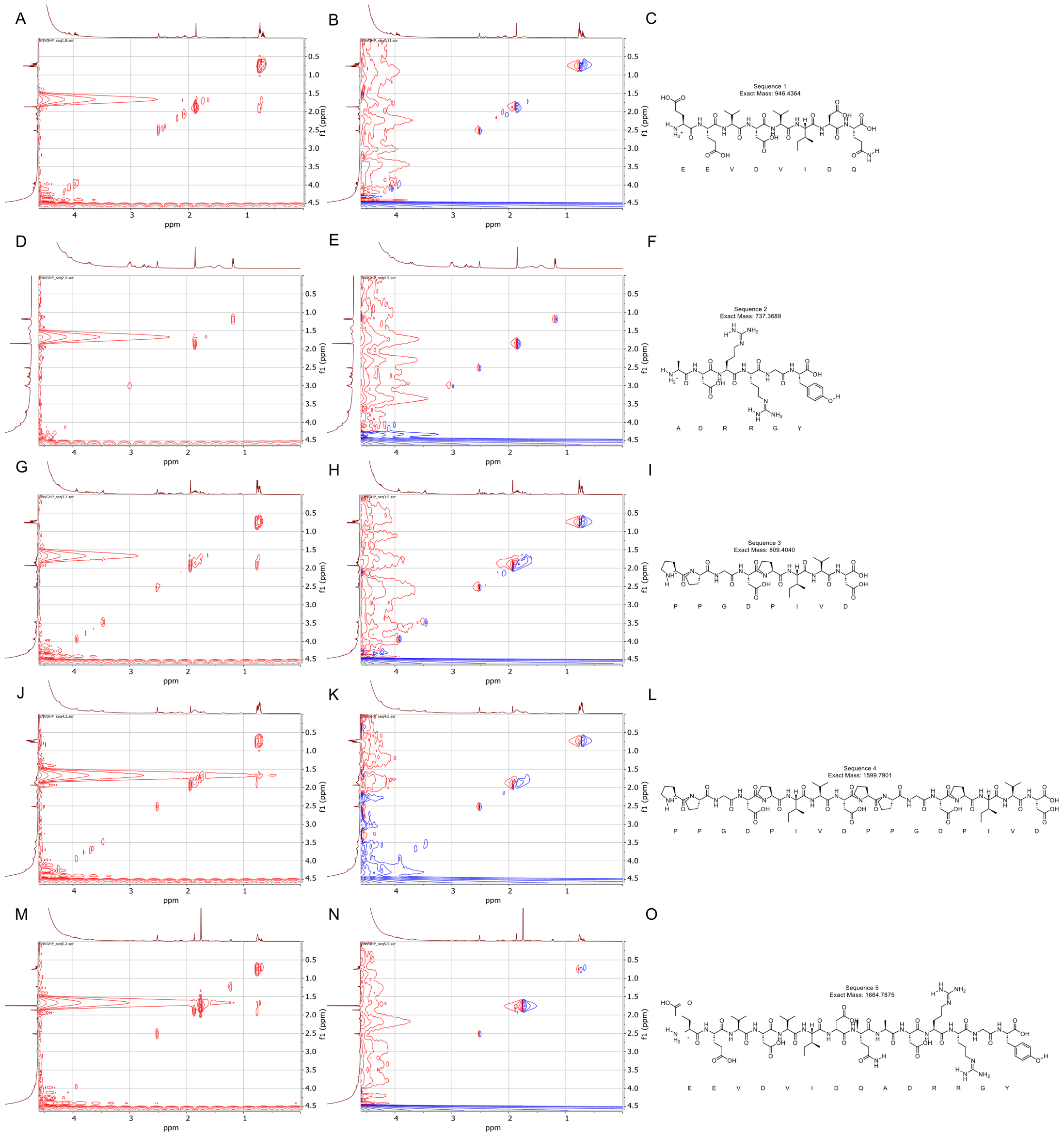

Fig. S7. NMR analysis of synthetic NPG peptide modules. (A, D, G, J, M) ^{1}H-^{1}H COSY spectra (0.0-4.6 ppm) of five representative synthetic NPG peptide sequences, showing resolved spin systems in the aliphatic region. **(B, E, H, K, N)** Corresponding NOESY spectra for each peptide, acquired in the same chemical shift range. No cross-peaks indicative of long-range correlations or stable secondary structure were observed in any sequence. **(C, F, I, L, O)** Molecular structures of peptides 1-5 with annotated monoisotopic $[M+H]^{+}$ masses, confirming sequence identity and purity.

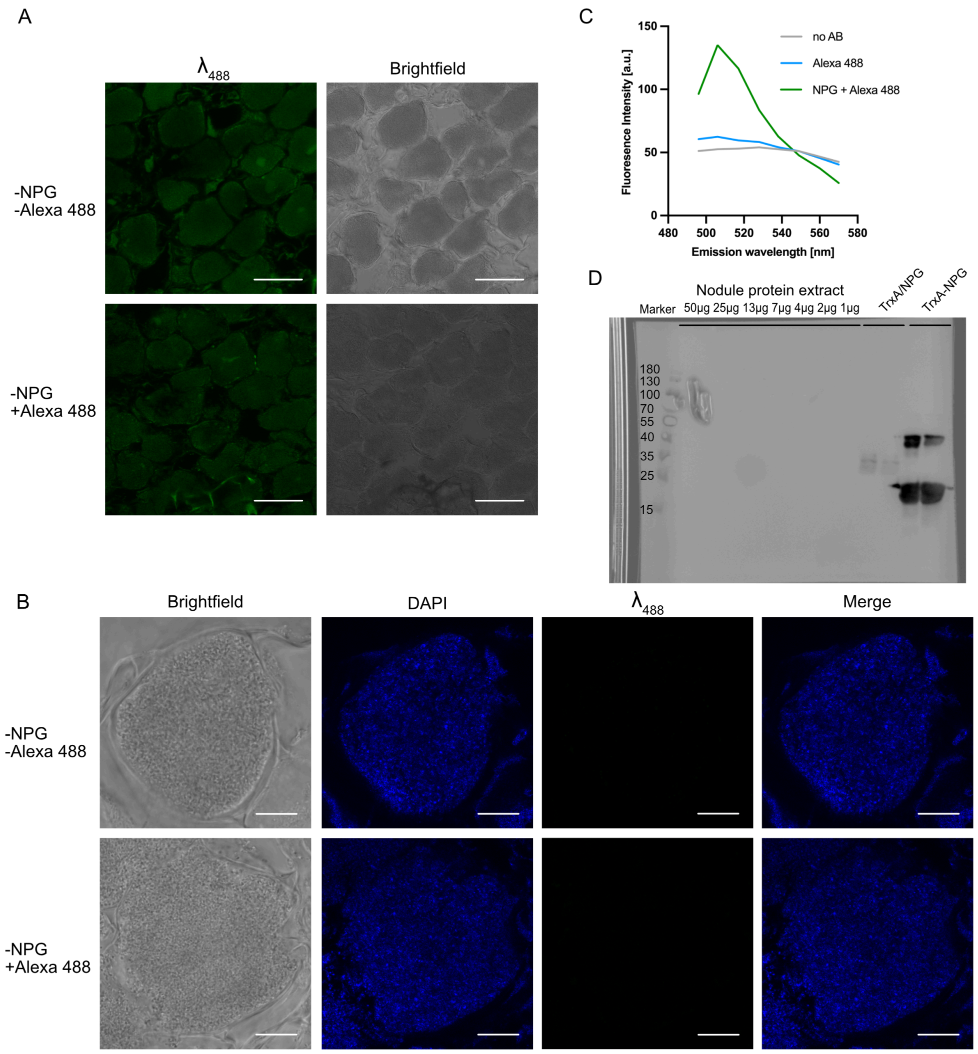

Fig. S8. Control immunofluorescence staining of *Robinia pseudoacacia* nodules. (A) Low-magnification view of cryosectioned nodule tissue (20 µm, 40× water objective, 4% formaldehyde fixation) imaged without primary antibody (Alexa Fluor 488 secondary only), showing background fluorescence and tissue structure. Scale bar, 50 µm. **(B)** High-magnification image (100× oil objective) of the same control condition, highlighting cellular and subcellular detail. Scale bar, 10 µm. **(C)** Emission spectra from lambda scanning (excitation 488 nm, 496-570 nm, 10 nm steps) under three conditions: no antibody, Alexa Fluor 488 secondary only, and full staining (anti-NPG primary + Alexa Fluor 488 secondary). Only full staining yielded a specific peak (500-530 nm), confirming signal specificity. **(D)** Western blot of nodule protein extracts (1-50 µg/lane) probed with anti-NPG antibodies. No bands were detected in plant tissue, excluding background recognition. Positive controls included purified recombinant proteins: TrxA/NPG (TEV-cleaved; ~15 kDa), monomeric NPG (~5 kDa), TrxA-NPG fusion (~20 kDa); all showing expected dimerization. Lanes loaded with 1 µg recombinant protein. Marker: PageRuler™ Prestained Protein Ladder (#26616, Thermo Scientific). These controls confirm antibody specificity and absence of cross-reactivity with plant proteins. Representative of two independent experiments.

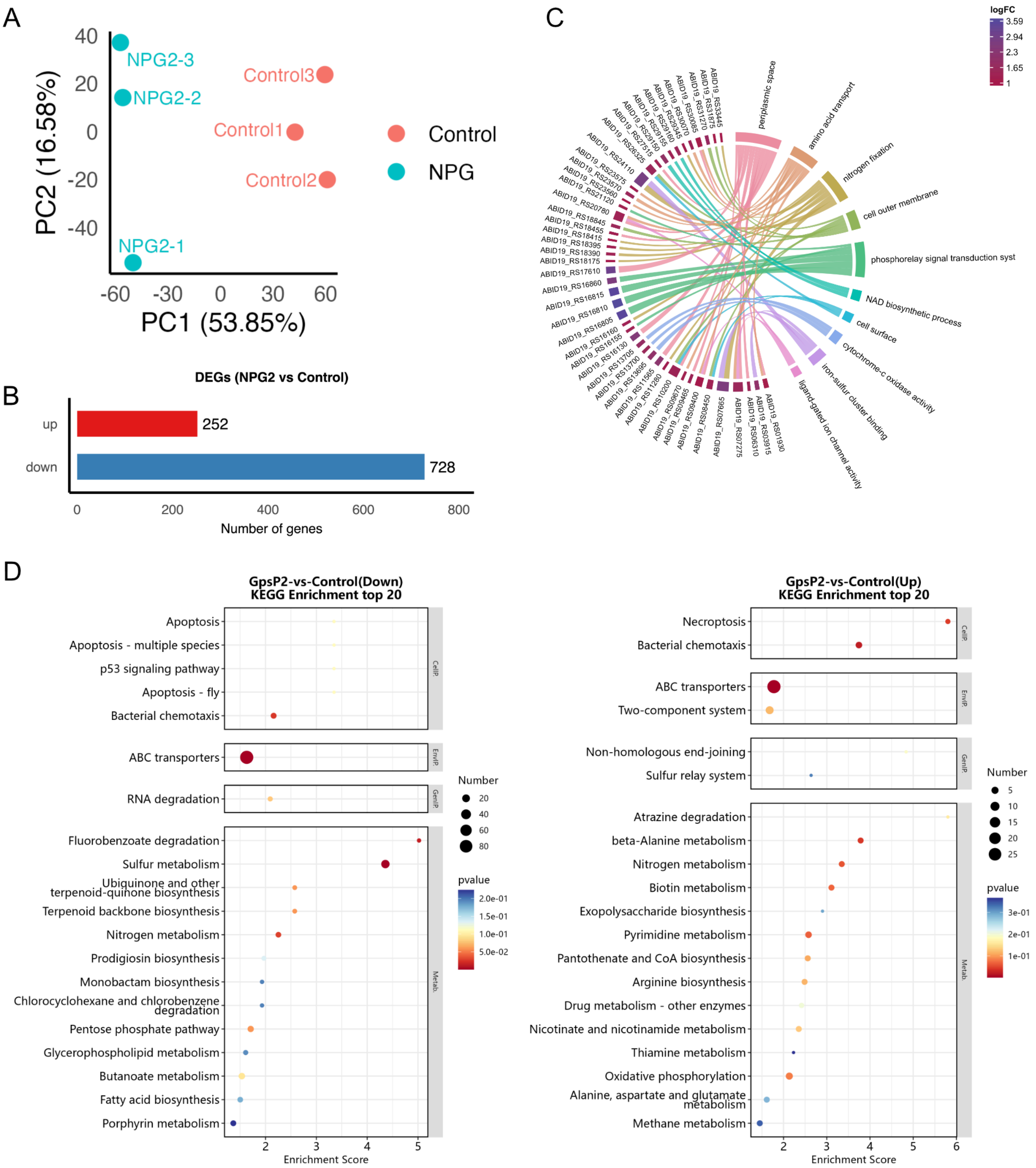

Fig. S9. Transcriptomic responses of *M. robiniae* to NPG2 exposure. (A) Principal component analysis (PCA) of RNA-seq data from *M. robiniae* cultures treated with 250 µM NPG2 for 2 hours (n = 3 biological replicates) and untreated controls (n = 3). **(B)** Counts of differentially expressed genes (DEGs) at q < 0.05 and |log2FC| ≥ 1, grouped as up- and downregulated. **(C)** Gene Ontology (GO) chord diagram linking significantly upregulated genes to enriched biological processes, showing connections across functional categories. **(D)** Kyoto Encyclopaedia of Genes and Genomes (KEGG) pathway enrichment plots, with significantly enriched downregulated pathways shown on the left and upregulated pathways on the right.

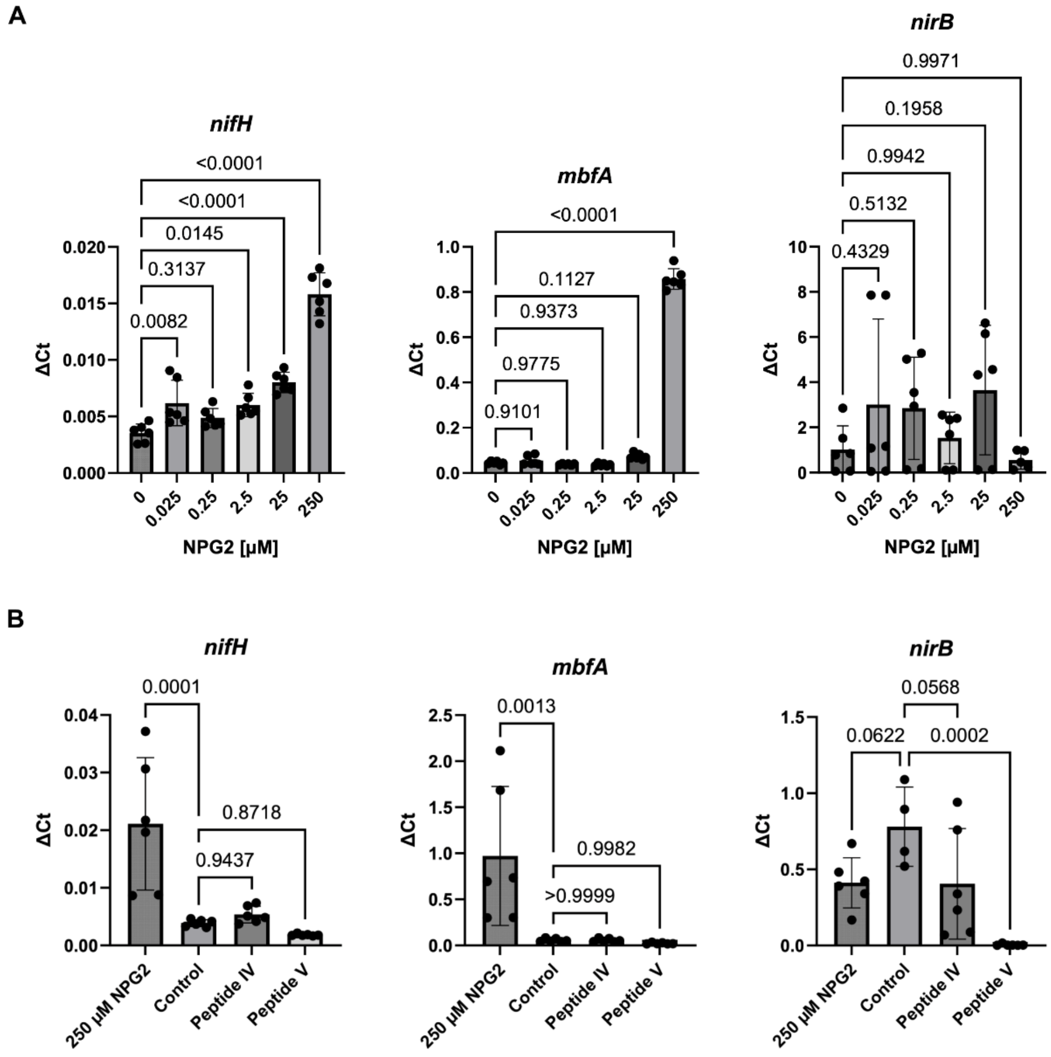

Fig. S10. Dose-dependent transcriptional responses of *M. robiniae* to NPG peptides assessed by RT–qPCR. (**A**) Relative transcript levels of representative NPG2-responsive genes following 2 h exposure to increasing concentrations of recombinant NPG2. Induction of *nifH* was detectable at 0.025 µM and increased with peptide concentration, reaching ~3-fold upregulation at 250 µM. *mbfA* showed no significant change at sub-micromolar concentrations and was strongly induced only at 250 µM. *nirB* exhibited a biphasic response, with modest, non-significant induction at low micromolar concentrations and mild repression at 250 µM. (**B**) Transcript levels following treatment with truncated NPG2 peptides corresponding to the N-terminal or repeat region (250 µM). Neither truncated peptide reproduced the transcriptional profile induced by full-length NPG2. Transcript levels were normalized to the housekeeping gene *rpsB* and/or *rpsU* using the ΔCq method and are shown relative to PBS-treated controls. Data represent mean ± s.d. from independent biological replicates, each analyzed in two technical replicates. Statistical significance was determined by ordinary one-way ANOVA.

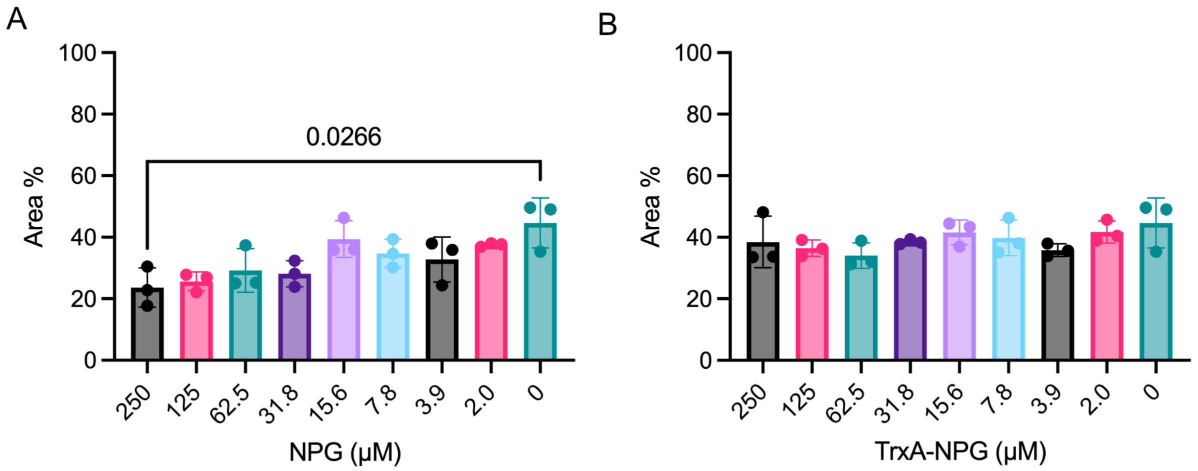

Fig. S11. Concentration-dependent growth inhibition by NPG2 on solid medium. *M. robiniae* DSM 100022 cultures grown in YEM broth to mid-log (OD_{600} = 0.4-0.5) were washed, adjusted to OD_{600} = 0.2, and incubated for 2 h at 28 °C with 0-250 µM peptide. After serial dilution, 100 µL aliquots were plated on YEM agar and incubated at 28 °C for 3-5 d; plate growth was quantified from scanned images as colony-covered area using Fiji v1.53. **(A)** Mature NPG2 (TEV-cleaved). **(B)** TrxA-NPG2 fusion protein. Points denote replicate means; bars show mean ± s.d. for n = 3 independent cultures. Mature NPG2 reduced plate coverage in a concentration-dependent manner, whereas the TrxA-NPG2 fusion had no detectable effect across the same range. Statistical significance was determined by Student's t-test (* = $P < 0.05$).

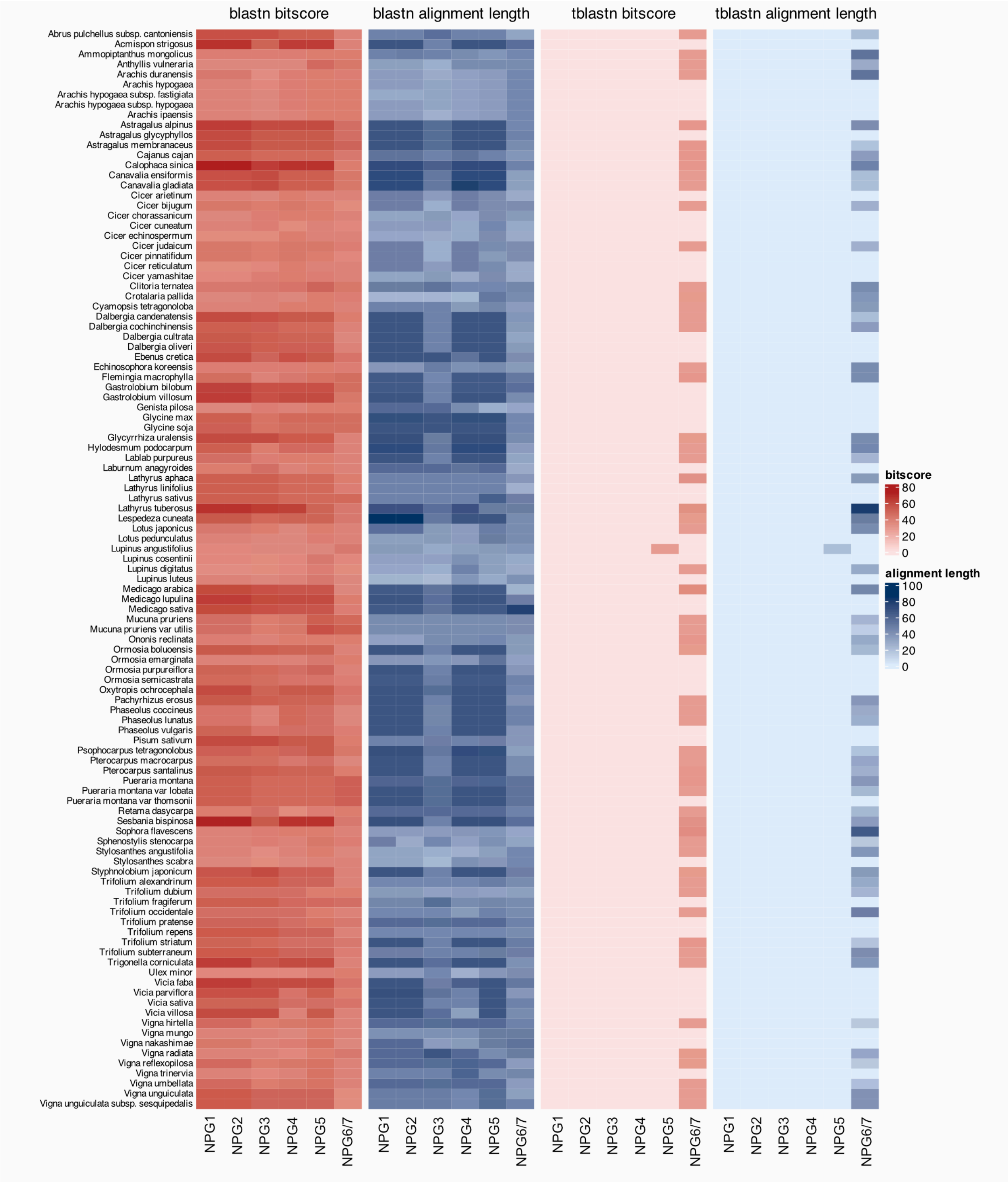

Fig. S12. BLAST alignment metrics of NPG homologous sequences across species. Heatmaps showing the maximum BLASTn and tBLASTn bitscores (red) and alignment lengths (blue) for NPG candidate sequences across all analyzed species. Most extended alignments correspond to N-terminal signal peptide sequences rather than NPG-specific regions.

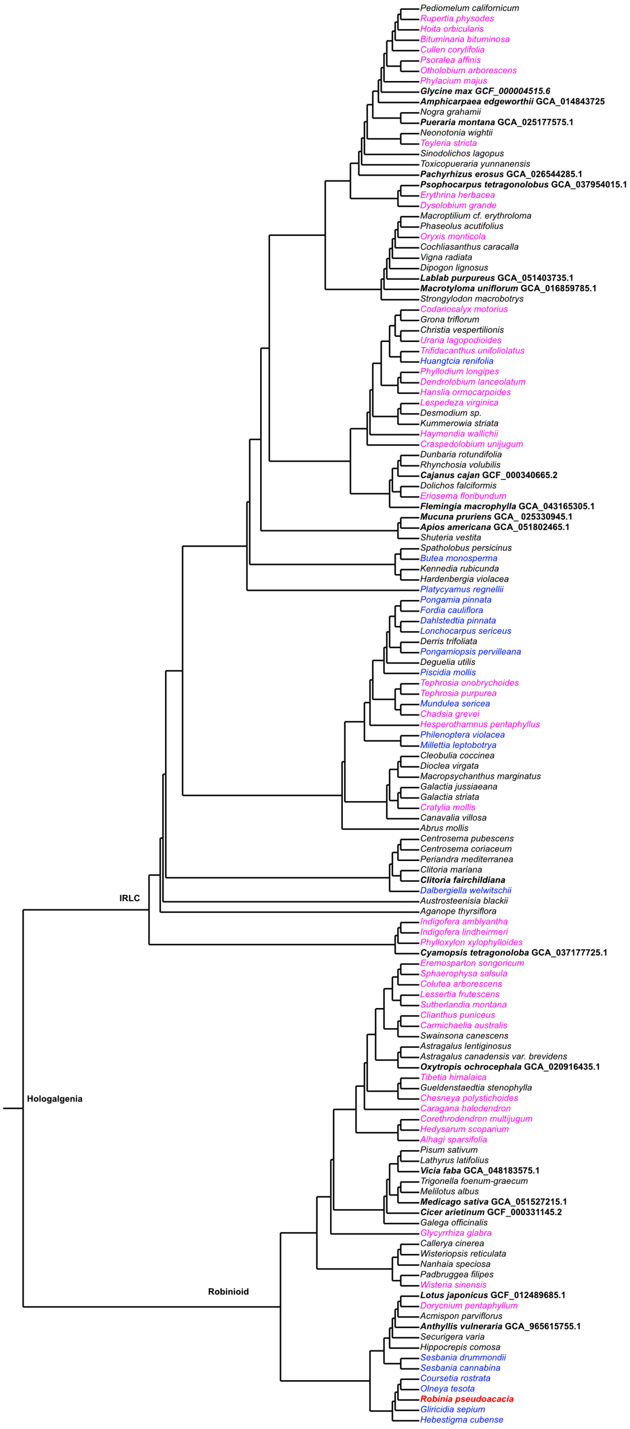

Fig. S13. Phylogeny of nitrogen-fixing plants. Maximum-likelihood phylogeny based on the Cai et al. (2025) Hologalegina dataset (OpenTree study OT_2372) . Tree-forming species are highlighted in blue, shrubs in magenta, with *R. pseudoacacia* distinctively highlighted in red. Species with available genome assemblies in NCBI are shown in bold, with the corresponding assembly accession numbers listed adjacent to taxon labels. Notably, *Robinia* is the only tree lineage within Hologalegina represented by genomic data, emphasizing the phylogenetic contrast between the NCR-containing IRLC and the NPG-containing *Robinia*. Bootstrap support values are provided for major nodes.

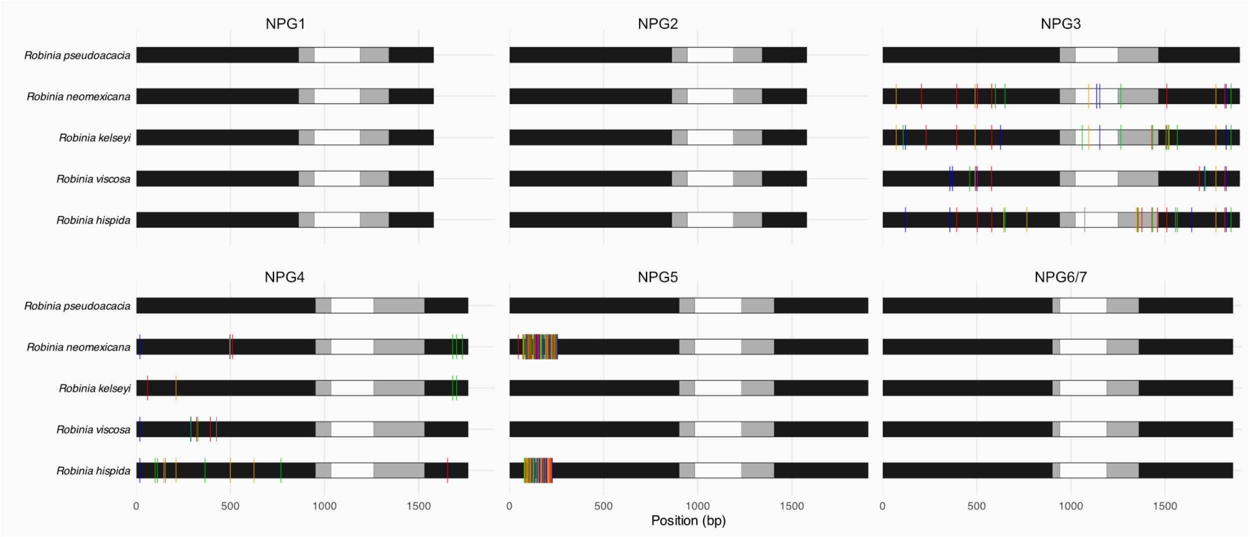

Fig. S14. Genomic distribution and sequence variation of NPG genes in *Robinia*. Short-read genomic data were mapped to the *R. pseudoacacia* reference assembly using BWA, processed with SAMtools, BEDTools, and BCFtools (*62*), and visualized in IGV (*72*). Horizontal bars indicate read coverage; coding sequences are shown in grey and introns in white. Vertical lines mark single-nucleotide variants, colored by the major allele (A, green; C, blue; G, orange; T, red). Reads mapped across all NPG loci, confirming their presence and genomic context.

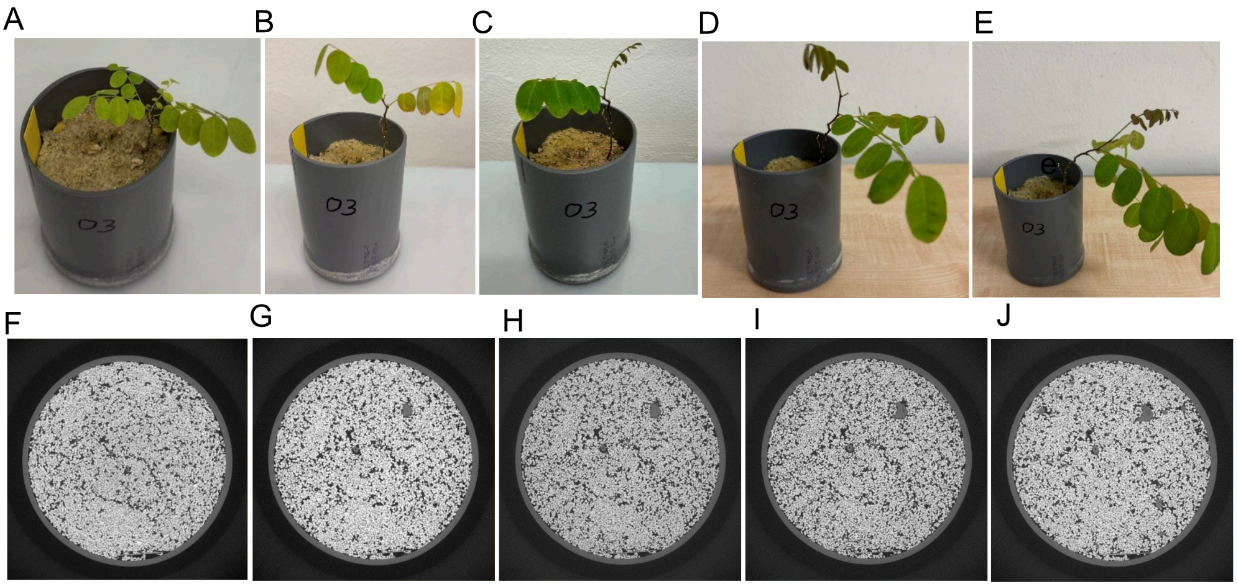

Fig. S15. Time series of plant growth and CT reconstructions of *Robinia pseudoacacia* nodulation. (A-E) Photographs of plants taken before inoculation (-4 d) and at 21 d, 33 d, 63 d, and 84 d after inoculation. **(F-J)** Corresponding μCT reconstructions of the same plants at identical time points, all showing the same slice (slice 747) to allow direct comparison of root and nodule development across the time course.

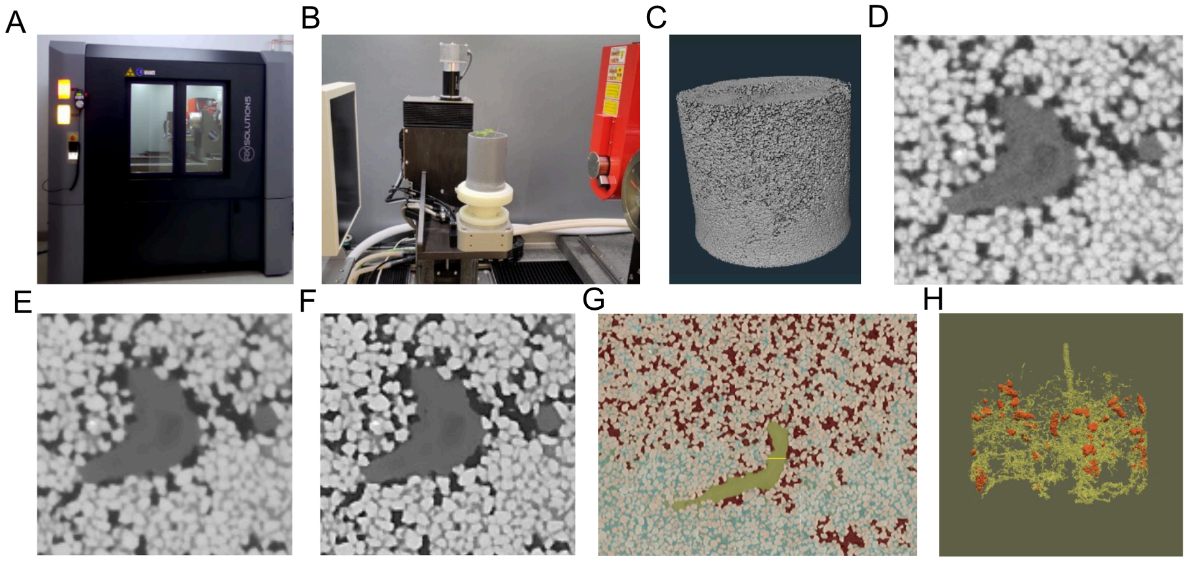

Fig. S16. Workflow for CT-based image processing of *Robinia* root systems. (A) X-ray CT scanning setup, **(B)** CT chamber with mounted pot, **(C)** scanned plant sample, **(D)** raw grayscale slice, **(E)** denoised image after Non-Local Means filtering, **(F)** edge-enhanced image after Unsharp Masking, **(G)** segmentation in Ilastik, and **(H)** segmented root phase with nodules highlighted in red.

Table S1. NPG peptide sequences and features in *Robinia pseudoacacia*.

Protein	Gene_id	AA_Sequence	Length	Similarity	Signal_Peptide	SP Length	Mature Seq	M Length	Glycine %	Proline %
**NPG1**	3G00089040	MGPKKASLILGLLVMILLLSSEATREEVDVIDQADRRGYPPGDPIVDPPGDPIVDPPGDPIGDPPGDPIGDPPIGGG	77	100	MGPKKASLILGLLVMILLLSSEATR	25	EEVDVIDQADRRGYPPGDPIVDPPGDPIVDPPGDPIGDPPGDPIGDPPIGGG	52	19.2	26.9
**NPG2**	3G00089840	MGPKKASLILGLLVMILLLSSEATREEVDVIDQADRRGYPPGDPIVDPPGDPIVDPPGDPIVDPPGDPIGDPPIGGG	77	98.7	MGPKKASLILGLLVMILLLSSEATR	25	EEVDVIDQADRRGYPPGDPIVDPPGDPIVDPPGDPIVDPPGDPIGDPPIGGG	52	17.3	26.9
**NPG3**	3G00089570	MGPKKASLILGLLVMILLLASEATREEVDVIDQVGDPPGSGNPIGDPPGEEDPIGDPPGEEDPIGDPPGEEDPIGDPPGGGAPIGDPPGGGSCGDLAAQTQNNTLNGCGGLNNGNI	116	56.9	MGPKKASLILGLLVMILLLASEATR	25	EEVDVIDQVGDPPGSGNPIGDPPGEEDPIGDPPGEEDPIGDPPGEEDPIGDPPGGGAPIGDPPGGGSCGDLAAQTQNNTLNGCGGLNNGNI	91	24.2	18.7
**NPG4**	3G00090040	MGLEKASLILGLLVMILLLSSEATREKVYGIDPPGGGHPIGEPPGSGGHPISEPPGSGGHPIGEPSGGHPIGEPPGGGPPIGGPLCGGTAQTLNNTLN	98	53.1	MGLEKASLILGLLVMILLLSSEATR	25	EKVYGIDPPGGGHPIGEPPGSGGHPISEPPGSGGHPIGEPSGGHPIGEPPGGGPPIGGPLCGGTAQTLNNTLN	73	30.1	21.9
**NPG5**	3G00089880	MGPKKASLILGLLVMTLLLSSEATREEDDVVEEETNCVGDAKYYGNPNPPSGDNRPGDGHDGPQGDFPGGGCGGVAAQTQNNTLN	85	47.6	MGPKKASLILGLLVMTLLLSSEATR	25	EEDDVVEETNCVGDAKYYGNPNPPSGDNRPGDGHDGPQGDFPGGGCGGVAAQTQNNTLN	60	20.0	10.0
**NPG6**	3G00090420	MILLLSSEATWEEVDVIEETNGVGDAKYYGNPHPPSGGKRRDPPSGVHGDPARGGGGGIAAQTQNKTLN	69	39	MILLLSSEA	9	TWEEVDVIEETNGVGDAKYYGNPHPPSGGKRRDPPSGVHGDPARGGGGGIAAQTQNKTLN	60	20.0	10.0
**NPG7**	3G00089080	MILLLSSEATWEEVDVIEETNGVGDAKYYGNPHPPSGGKRRDPPSGVHGDPARGGGGGIAAQTQNKTLN	69	39	MILLLSSEA	9	TWEEVDVIEETNGVGDAKYYGNPHPPSGGKRRDPPSGVHGDPARGGGGGIAAQTQNKTLN	60	20.0	10.0

Table S2. Identified peptides of NPGs.

protein	identified peptide[1]	endoprotease	unique peptide[2]	number of replicates[3]	MS-instrument (ion fragmentation)	Intensities
NPG1	DPIVDPPGDPIG*	AspN	no	2	Fusion (HCD)	10,488,000 / 15,410,000
	DPIVDPPGDPIVDPPG*	AspN	no	2	Orbitrap Velos Pro (CID), Fusion (HCD)	10,300,000 / 27,578,000
	DPIVDPPGDPIVDPPGDPIG*	AspN	no	2	Orbitrap Velos Pro (CID), Fusion (HCD)	5,198,100 / 7,347,600
	DPPGDPIVDPPGDPIG*	AspN	no	1	Orbitrap Velos Pro (CID)	496,410
NPG2	DPIVDPPGDPIG*	AspN	no	2	Fusion (HCD)	10,488,000 / 15,410,000
	DPIVDPPGDPIVDPPG*	AspN	no	2	Orbitrap Velos Pro (CID), Fusion (HCD)	10,300,000 / 27,578,000
	DPIVDPPGDPIVDPPGDPIG*	AspN	no	2	Orbitrap Velos Pro (CID), Fusion (HCD)	5,198,100 / 7,347,600
	DPPGDPIVDPPGDPIG*	AspN	no	1	Orbitrap Velos Pro (CID)	496,410
	DPIVDPPGDPIVDPPGDPIVDPPG	AspN	yes	2	Orbitrap Velos Pro (CID)	750,140 / 149,790
	DPPGDPIVDPPGDPIV	AspN/α-lytic protease	yes	1/1	Fusion (HCD)	6,988,900 / 795,200
NPG3	DPPGSGNPIGDPPGEEDPIGDPPGEE	AspN	yes	1	Orbitrap Velos Pro (CID)	193,720
	DPIGDPPGEEDPIGDPPGEEDPIG	AspN	yes	1	Orbitrap Velos Pro (CID)	610,800

[1] Peptides that are shared by the two proteins are marked with an asterisk (*).
[2] This indicates whether the identified peptide is unique to the particular protein.
[3] Number of replicates where the peptide was identified.

Table S3. DeepLoc2.1 localization prediction.

Protein	Localizations	Signals	Membrane types	Cyto-plasm	Nucleus	Extra-cellular	Cell-membrane	Mito-chondrion	Plastid	Endoplasmic reticulum	Lysosome/Vacuole	Golgi apparatus	Peroxisome	Peripheral	Transmembrane	Lipid anchor	Soluble
**NPG1**	Extracellular	Signal peptide	Soluble	0.049	0.112	0.830	0.177	0.009	0.101	0.351	0.326	0.318	0.057	0.105	0.050	0.120	0.907
**NPG2**	Extracellular	Signal peptide	Soluble	0.046	0.113	0.827	0.183	0.008	0.099	0.355	0.335	0.310	0.054	0.090	0.052	0.131	0.908
**NPG3**	Extracellular	Signal peptide	Soluble	0.090	0.135	0.811	0.317	0.009	0.089	0.218	0.442	0.319	0.045	0.205	0.038	0.130	0.902
**NPG4**	Extracellular	Signal peptide	Soluble	0.041	0.095	0.827	0.253	0.006	0.156	0.315	0.368	0.252	0.036	0.157	0.092	0.073	0.836
**NPG5**	Extracellular	Signal peptide	Soluble	0.039	0.127	0.765	0.215	0.007	0.099	0.296	0.353	0.153	0.038	0.147	0.068	0.138	0.797
**NPG6**	Nucleus		Soluble	0.348	0.494	0.423	0.125	0.199	0.113	0.284	0.050	0.042	0.006	0.157	0.158	0.316	0.843
**NPG7**	Nucleus		Soluble	0.348	0.494	0.423	0.125	0.199	0.113	0.284	0.050	0.042	0.006	0.157	0.158	0.316	0.843

Table S4. pLDDT scores for mature NPG1 (left) NPG2 (right).

Position in full chain	Residue name	pLDDT	Residue name	pLDDT
26	GLU	69.27	GLU	67.86
27	GLU	66.11	GLU	64.4
28	VAL	68.05	VAL	66.36
29	ASP	61.91	ASP	64.93
30	VAL	68.08	VAL	68.05
31	ILE	64.46	ILE	63.97
32	ASP	64.78	ASP	66.28
33	GLN	66.7	GLN	71.11
34	ALA	66.3	ALA	67.5
35	ASP	64.93	ASP	66.11
36	ARG	68.16	ARG	68.68
37	ARG	67.76	ARG	68.17
38	GLY	63.83	GLY	65.14
39	TYR	58.33	TYR	59.92
40	PRO	62.33	PRO	62.2
41	PRO	64.15	PRO	64.28
42	GLY	62.8	GLY	62.1
43	ASP	59.51	ASP	60.3
44	PRO	63.9	PRO	64.12
45	ILE	67.16	ILE	67.08
46	VAL	67.75	VAL	66.28
47	ASP	64.46	ASP	65.96
48	PRO	69.38	PRO	70.78
49	PRO	69.66	PRO	70.96
50	GLY	66.5	GLY	68.42
51	ASP	62.13	ASP	63.73
52	PRO	64.2	PRO	65.63
53	ILE	67.97	ILE	67.38
54	VAL	63.55	VAL	64.85
55	ASP	62.29	ASP	63.85
56	PRO	65.75	PRO	67.06
57	PRO	64.57	PRO	66.85
58	GLY	63.14	GLY	65.7
59	ASP	60.7	ASP	63.83
60	PRO	60.94	PRO	64.43
61	ILE	64.58	ILE	66.59
62	GLY	61.44	VAL	63.06
63	ASP	61.74	ASP	65.15
64	PRO	63.98	PRO	68.35
65	PRO	64.89	PRO	67.95
66	GLY	64.51	GLY	66.71
67	ASP	64.93	ASP	67.83
68	PRO	66.32	PRO	67.84
69	ILE	69.85	ILE	71.16
70	GLY	68.02	GLY	70.1
71	ASP	69.53	ASP	71.82
72	PRO	73.38	PRO	74.95
73	PRO	74.06	PRO	75.06
74	ILE	72.46	ILE	74.89
75	GLY	73.15	GLY	74.68
76	GLY	64.18	GLY	65.95
77	GLY	62.83	GLY	64.21

Table S5. Synthesized peptides of NPG2.

Peptide	Sequence	Length	Purity	Modifications	TFA_Removal	Notes
Seq1	EEVDVIDQ	8	>95%	N-terminal acetylation; C-terminal amidation	Yes (HCl salt)	N-terminal region after signal peptide; most conserved
Seq2	ADRRGY	6	>95%	N-terminal acetylation; C-terminal amidation	Yes (HCl salt)	Highly charged region (NPG1/2 only); possible catalytic or membrane interaction
Seq3	PPGDPIVD	8	>95%	N-terminal acetylation; C-terminal amidation	Yes (HCl salt)	Distinctive motif repeated across NPG family
Seq4	PPGDPIVDPPGDPIVD	16	>95%	N-terminal acetylation; C-terminal amidation	Yes (HCl salt)	Duplication of motif to test chaining effect (highest priority)
Seq5	EEVDVIDQADRRGY	14	>95%	N-terminal acetylation; C-terminal amidation	Yes (HCl salt)	Combination of Seq1+Seq2 to cover full N-terminal region

Table S6. Cloning primers used in this study.

Name	Sequence
SDM_S_NPG1.for	tttgaaaaaggaggatctGAAGAAGTTGATGTTATTGATC
SDM_S_NPG1.rev	ctgcggatggctccaggaTTGGAAGTACAGGTTTTC
F_aNPG1.FOR	ccaatccACACGGGAAGAAGTTGATGTTATTGAT
V_aNPG1.REV	TCCCGTGTggattggaagtacaggttttcctcgatccc
V_aNPG1.FOR	GTGGTTGAtgactcgagcaccaccac
F_aNPG1.REV	ctcgagtcaTCAACCACCACCTATAGGAGGAT
F_aNPG2.FOR	ccaatccACACGGGAAGAAGTTGATGTTATTGAT
V_aNPG2.REV	TCCCGTGTggattggaagtacaggttttcctcgatccc
V_aNPG2.FOR	GTGGTTGAtgactcgagcaccaccac
F_aNPG2.REV	ctcgagtcaTCAACCACCACCTATAGGAGGAT
F1.pCV_NPG1.FOR	GTTGCTGGCGTTTTTCCATAGGCT
F1.pCV_NPG1.REV	TCTTCGGACCCATGATCTCGAGCGTGTCCTCT
F2.pCV_NPG1.FOR	ACGCTCGAGATCATGGGTCCGAAGAAAGCGT
F2.pCV_NPG1.REV	CCATCCTAGGCACACCACCACCTATAGGAGGATCAC
V.pCV_NPG1.FOR	TATAGGTGGTGGTGTGCCTAGGATGGTGAGCAAG
V.pCV_NPG1.REV	GAGCCTATGGAAAAACGCCAGCAACG
F1.pCV_NPG6.FOR	GTTGCTGGCGTTTTTCCATAGGCT
F1.pCV_NPG6.REV	GCAGAAGAATCATGATCTCGAGCGTGTCCTCT
F2.pCV_NPG6.FOR	ACGCTCGAGATCATGATTCTTCTGCTTTCCTCAGAGGC
F2.pCV_NPG6.REV	CCATCCTAGGCACGTTGAGAGTCTTGTTCTGAGTTTGTGC
V.pCV_NPG6.FOR	AAGACTCTCAACGTGCCTAGGATGGTGAGCAAG
V.pCV_NPG6.REV	GAGCCTATGGAAAAACGCCAGCAACG

Table S7. qPCR primers used in this study.

Primer name	Sequence 5'->3'
nifH FWD Set 3	CAT ACT GGA TCA CCG ACA TCT TC
nifH REV Set 3	TGG CTT CCA GGC TCA ATT C
mbfA FWD Set 2	GGG CTT GCG CTC GTA ATA
mbfA REV Set 2	GAC TCG CTG ATA GAG CTT CAC
nirB.for	AGT CGT CGG CAT ATT CGT CC
nirB.rev	CAT GCA GGA GCT GGA ATG GA
rpsU.for	GGT ACT CGT CCG CGA TAA CA
rpsU.rev	CCG ACG GCT TCT CGT AAT GT
rpsB.for	GCC CGA TCT GAT GTT CGT GA
rpsB.rev	GAT CTT GTC CGG ATC GCA GT

Movie S1. Pre-inoculation root morphology (-4 dpi). Time-lapse imaging of root architecture four days prior to inoculation with *Mesorhizobium robiniae*, showing baseline cellular organization and absence of nodulation structures.

Movie S2. Early nodule development (21 dpi). Dynamic progression of nodule formation 21 days after inoculation with *M. robiniae*, highlighting initial cortical cell divisions and emerging nodule primordia.

Movie S3. Intermediate nodule maturation (33 dpi). Time-resolved imaging at 33 days post-inoculation illustrating expansion and structural differentiation of developing nodules.

Movie S4. Late-stage nodulation (63 dpi). Advanced nodulation phenotype at 63 days after inoculation with *M. robiniae*, demonstrating mature nodule architecture and tissue organization.

REFERENCES

1. J. N. Galloway, A. R. Townsend, J. W. Erisman, M. Bekunda, Z. Cai, J. R. Freney, L. A. Martinelli, S. P. Seitzinger, M. A. Sutton, Transformation of the nitrogen cycle: Recent trends, questions, and potential solutions. *Science* **320**, 889–892 (2008).

2. G. E. Oldroyd, J. D. Murray, P. S. Poole, J. A. Downie, The rules of engagement in the legume-rhizobial symbiosis. *Annu. Rev. Genet.* **45**, 119–144 (2011).

3. D. F. Herridge, M. B. Peoples, R. M. Boddey, Global inputs of biological nitrogen fixation in agricultural systems. *Plant and Soil* **311**, 1–18 (2008).

4. P. Czernic, D. Gully, F. Cartieaux, L. Moulin, I. Guefrachi, D. Patrel, O. Pierre, J. Fardoux, C. Chaintreuil, P. Nguyen, F. Gressent, C. Da Silva, J. Poulain, P. Wincker, V. Rofidal, S. Hem, Q. Barriere, J. F. Arrighi, P. Mergaert, E. Giraud, Convergent evolution of endosymbiont differentiation in Dalbergioid and inverted repeat-lacking clade legumes mediated by nodule-specific cysteine-rich peptides. *Plant Physiol.* **169**, 1254–1265 (2015).

5. P. Mergaert, T. Uchiumi, B. Alunni, G. Evanno, A. Cheron, O. Catrice, A. E. Mausset, F. Barloy-Hubler, F. Galibert, A. Kondorosi, E. Kondorosi, Eukaryotic control on bacterial cell cycle and differentiation in the Rhizobium-legume symbiosis. *Proc. Natl. Acad. Sci. U.S.A.* **103**, 5230–5235 (2006).

6. W. Van de Velde, G. Zehirov, A. Szatmari, M. Debreczeny, H. Ishihara, Z. Kevei, A. Farkas, K. Mikulass, A. Nagy, H. Tiricz, B. Satiat-Jeunemaitre, B. Alunni, M. Bourge, K. Kucho, M. Abe, A. Kereszt, G. Maroti, T. Uchiumi, E. Kondorosi, P. Mergaert, Plant peptides govern terminal differentiation of bacteria in symbiosis. *Science* **327**, 1122–1126 (2010).

7. J. I. Sprent, *Legume Nodulation: A Global Perspective* (Wiley-Blackwell, 2009).

8. J. I. Sprent, Evolving ideas of legume evolution and diversity: A taxonomic perspective on the occurrence of nodulation. *New Phytol.* **174**, 11–25 (2007).

9. J. Montiel, A. Szucs, I. Z. Boboescu, V. D. Gherman, E. Kondorosi, A. Kereszt, Terminal bacteroid differentiation is associated with variable morphological changes in legume species belonging to the inverted repeat-lacking clade. *Mol. Plant Microbe Interact.* **29**, 210–219 (2016).

10. D. Wang, J. Griffitts, C. Starker, E. Fedorova, E. Limpens, S. Ivanov, T. Bisseling, S. Long, A nodule-specific protein secretory pathway required for nitrogen-fixing symbiosis. *Science* **327**, 1126–1129 (2010).

11. E. Kondorosi, P. Mergaert, A. Kereszt, A paradigm for endosymbiotic life: Cell differentiation of Rhizobium bacteria provoked by host plant factors. *Annu. Rev. Microbiol.* **67**, 611–628 (2013).

12. R. Oono, I. Schmitt, J. I. Sprent, R. F. Denison, Multiple evolutionary origins of legume traits leading to extreme rhizobial differentiation. *New Phytol.* **187**, 508–520 (2010).

13. L. Cai, D. Cardoso, L. G. Tressel, C. Lee, B. Shrestha, I. S. Choi, H. C. de Lima, L. P. de Queiroz, T. A. Ruhlman, R. K. Jansen, M. F. Wojciechowski, Well-resolved phylogeny supports repeated evolution of keel flowers as a synergistic contributor to papilionoid legume diversification. *New Phytol.* **247**, 369–387 (2025).

14. B. Hu, M. Messerer, G. Haberer, T. Lux, V. Marosi, K. F. X. Mayer, K. D. Oliphant, D. Kaufholdt, J. Schulze, L. S. Kreth, J. Jurgeleit, R. Geffers, R. Hansch, H. Rennenberg, Genomic and transcriptomic insights into legume-rhizobia symbiosis in the nitrogen-fixing tree Robinia pseudoacacia. *New Phytol.* **246**, 2522–2536 (2025).

15. Y. Kaneko, E. H. Newcomb, Specialization for ureide biogenesis in the root nodules of black locust (Robinia pseudoacacia L.), an amide exporter. *Protoplasma* **157**, 102–111 (1990).

16. L. R. Boring, W. T. Swank, The role of black locust (Robinia pseudo-acacia) in forest succession. *J. Ecol.* **72**, 749 (1984).

17. A. Cierjacks, I. Kowarik, J. Joshi, S. Hempel, M. Ristow, M. von der Lippe, E. Weber, Biological Flora of the British Isles: *Robinia pseudoacacia*. *J. Ecol.* **101**, 1623–1640 (2013).

18. R. Liu, K. D. Oliphant, Z. Xia, Z. Chen, R. Hou, R. Zhang, T. Peng, R. Hansch, D. Wang, H. Rennenberg, B. Hu, Rhizobial symbiosis modulates mercury accumulation and metabolic adaptation under hydrological extremes. *J. Hazard. Mater.* **495**, 139141 (2025).

19. B. Hu, M. Zhou, M. Dannenmann, G. Saiz, J. Simon, S. Bilela, X. Liu, L. Hou, H. Chen, S. Zhang, K. Butterbach-Bahl, H. Rennenberg, Comparison of nitrogen nutrition and soil carbon status of afforested stands established in degraded soil of the Loess Plateau, China. *For. Ecol. Manage.* **389**, 46–58 (2017).

20. S. Okamoto, E. Ohnishi, S. Sato, H. Takahashi, M. Nakazono, S. Tabata, M. Kawaguchi, Nod factor/nitrate-induced CLE genes that drive HAR1-mediated systemic regulation of nodulation. *Plant Cell Physiol.* **50**, 67–77 (2009).

21. T. Laloum, S. De Mita, P. Gamas, M. Baudin, A. Niebel, CCAAT-box binding transcription factors in plants: Y so many? *Trends Plant Sci.* **18**, 157–166 (2013).

22. G. Schroder, M. Fruhling, A. Puhler, A. M. Perlick, The temporal and spatial transcription pattern in root nodules of Vicia faba nodulin genes encoding glycine-rich proteins. *Plant Mol. Biol.* **33**, 113–123 (1997).

23. Z. Kevei, J. M. Vinardell, G. B. Kiss, A. Kondorosi, E. Kondorosi, Glycine-rich proteins encoded by a nodule-specific gene family are implicated in different stages of symbiotic nodule development in Medicago spp. *Mol. Plant Microbe Interact.* **15**, 922–931 (2002).

24. A. Kereszt, P. Mergaert, J. Montiel, G. Endre, É. Kondorosi, Impact of plant peptides on symbiotic nodule development and functioning. *Front. Plant Sci.* **9**, 1026 (2018).

25. N. D. Young, F. Debelle, G. E. Oldroyd, R. Geurts, S. B. Cannon, M. K. Udvardi, V. A. Benedito, K. F. Mayer, J. Gouzy, H. Schoof, Y. Van de Peer, S. Proost, D. R. Cook, B. C. Meyers, M. Spannagl, F. Cheung, S. De Mita, V. Krishnakumar, H. Gundlach, S. Zhou, J. Mudge, A. K. Bharti, J. D. Murray, M. A. Naoumkina, B. Rosen, K. A. Silverstein, H. Tang, S. Rombauts, P. X. Zhao, P. Zhou, V. Barbe, P. Bardou, M. Bechner, A. Bellec, A. Berger, H. Berges, S. Bidwell, T. Bisseling, N. Choisne, A. Couloux, R. Denny, S. Deshpande, X. Dai, J. J.

Doyle, A. M. Dudez, A. D. Farmer, S. Fouteau, C. Franken, C. Gibelin, J. Gish, S. Goldstein, A. J. Gonzalez, P. J. Green, A. Hallab, M. Hartog, A. Hua, S. J. Humphray, D. H. Jeong, Y. Jing, A. Jocker, S. M. Kenton, D. J. Kim, K. Klee, H. Lai, C. Lang, S. Lin, S. L. Macmil, G. Magdelenat, L. Matthews, J. McCorrison, E. L. Monaghan, J. H. Mun, F. Z. Najar, C. Nicholson, C. Noirot, M. O'Bleness, C. R. Paule, J. Poulain, F. Prion, B. Qin, C. Qu, E. F. Retzel, C. Riddle, E. Sallet, S. Samain, N. Samson, I. Sanders, O. Saurat, C. Scarpelli, T. Schiex, B. Segurens, A. J. Severin, D. J. Sherrier, R. Shi, S. Sims, S. R. Singer, S. Sinharoy, L. Sterck, A. Viollet, B. B. Wang, K. Wang, M. Wang, X. Wang, J. Warfsmann, J. Weissenbach, D. D. White, J. D. White, G. B. Wiley, P. Wincker, Y. Xing, L. Yang, Z. Yao, F. Ying, J. Zhai, L. Zhou, A. Zuber, J. Denarie, R. A. Dixon, G. D. May, D. C. Schwartz, J. Rogers, F. Quetier, C. D. Town, B. A. Roe, The Medicago genome provides insight into the evolution of rhizobial symbioses. *Nature* **480**, 520–524 (2011).

26. S. B. Cannon, A. Mitra, A. Baumgarten, N. D. Young, G. May, The roles of segmental and tandem gene duplication in the evolution of large gene families in *Arabidopsis thaliana*. *BMC Plant Biol.* **4**, 10 (2004).

27. S. Roy, S. Saxena, A. Sinha, A. K. Nandi, DORMANCY/AUXIN ASSOCIATED FAMILY PROTEIN 2 of Arabidopsis thaliana is a negative regulator of local and systemic acquired resistance. *J. Plant Res.* **133**, 409–417 (2020).

28. A. Puppo, K. Groten, F. Bastian, R. Carzaniga, M. Soussi, M. M. Lucas, M. R. de Felipe, J. Harrison, H. Vanacker, C. H. Foyer, Legume nodule senescence: Roles for redox and hormone signalling in the orchestration of the natural aging process. *New Phytol.* **165**, 683–701 (2005).

29. J. Abramson, J. Adler, J. Dunger, R. Evans, T. Green, A. Pritzel, O. Ronneberger, L. Willmore, A. J. Ballard, J. Bambrick, S. W. Bodenstein, D. A. Evans, C. C. Hung, M. O'Neill, D. Reiman, K. Tunyasuvunakool, Z. Wu, A. Zemgulyte, E. Arvaniti, C. Beattie, O. Bertolli, A. Bridgland, A. Cherepanov, M. Congreve, A. I. Cowen-Rivers, A. Cowie, M. Figurnov, F. B. Fuchs, H. Gladman, R. Jain, Y. A. Khan, C. M. R. Low, K. Perlin, A. Potapenko, P. Savy, S. Singh, A. Stecula, A. Thillaisundaram, C. Tong, S. Yakneen, E. D. Zhong, M. Zielinski, A. Zidek, V.

Bapst, P. Kohli, M. Jaderberg, D. Hassabis, J. M. Jumper, Accurate structure prediction of biomolecular interactions with AlphaFold 3. *Nature* **630**, 493–500 (2024).

30. A. Omidi, M. H. Moller, N. Malhis, J. M. Bui, J. Gsponer, AlphaFold-Multimer accurately captures interactions and dynamics of intrinsically disordered protein regions. *Proc. Natl. Acad. Sci. U.S.A.* **121**, e2406407121 (2024).

31. D. Piovesan, A. M. Monzon, S. C. E. Tosatto, Intrinsic protein disorder and conditional folding in AlphaFoldDB. *Protein Sci.* **31**, e4466 (2022).

32. L. Mularoni, A. Ledda, M. Toll-Riera, M. M. Alba, Natural selection drives the accumulation of amino acid tandem repeats in human proteins. *Genome Res.* **20**, 745–754 (2010).

33. R. Oono, R. F. Denison, Comparing symbiotic efficiency between swollen versus nonswollen rhizobial bacteroids. *Plant Physiol.* **154**, 1541–1548 (2010).

34. S. Wienkoop, G. Saalbach, Proteome analysis. Novel proteins identified at the peribacteroid membrane from Lotus japonicus root nodules. *Plant Physiol.* **131**, 1080–1090 (2003).

35. R. Dixon, D. Kahn, Genetic regulation of biological nitrogen fixation. *Nat. Rev. Microbiol.* **2**, 621–631 (2004).

36. I. J. Schalk, L. Guillon, Fate of ferrisiderophores after import across bacterial outer membranes: Different iron release strategies are observed in the cytoplasm or periplasm depending on the siderophore pathways. *Amino Acids* **44**, 1267–1277 (2013).

37. S. A. Cervantes-Perez, P. Zogli, S. Amini, S. Thibivilliers, S. Tennant, M. S. Hossain, H. Xu, I. Meyer, A. Nooka, P. Ma, Q. Yao, M. J. Naldrett, A. Farmer, O. Martin, S. Bhattacharya, J. Klaver, M. Libault, Single-cell transcriptome atlases of soybean root and mature nodule reveal new regulatory programs that control the nodulation process. *Plant Commun.* **5**, 100984 (2024).

38. B. Roux, N. Rodde, M. F. Jardinaud, T. Timmers, L. Sauviac, L. Cottret, S. Carrere, E. Sallet, E. Courcelle, S. Moreau, F. Debelle, D. Capela, F. de Carvalho-Niebel, J. Gouzy, C. Bruand, P.

Gamas, An integrated analysis of plant and bacterial gene expression in symbiotic root nodules using laser-capture microdissection coupled to RNA sequencing. *Plant J.* **77**, 817–837 (2014).

39. A. Gavrin, B. N. Kaiser, D. Geiger, S. D. Tyerman, Z. Wen, T. Bisseling, E. E. Fedorova, Adjustment of host cells for accommodation of symbiotic bacteria: Vacuole defunctionalization, HOPS suppression, and TIP1g retargeting in Medicago. *Plant Cell* **26**, 3809–3822 (2014).

40. A. Mangeon, R. M. Junqueira, G. Sachetto-Martins, Functional diversity of the plant glycine-rich proteins superfamily. *Plant Signal. Behav.* **5**, 99–104 (2010).

41. P. Mergaert, K. Nikovics, Z. Kelemen, N. Maunoury, D. Vaubert, A. Kondorosi, E. Kondorosi, A novel family in Medicago truncatula consisting of more than 300 nodule-specific genes coding for small, secreted polypeptides with conserved cysteine motifs. *Plant Physiol.* **132**, 161–173 (2003).

42. M. Song, Y. Zhou, G. Li, A. S. Barashkova, E. A. Rogozhin, W. Chang, Peptides in plant-microbe interactions: Functional diversity and pharmacological applications. *Cell Surf.* **13**, 100145 (2025).

43. R. M. Lima, B. B. Rathod, H. Tiricz, D. H. O. Howan, M. A. Al Bouni, S. Jenei, E. Timar, G. Endre, G. K. Toth, E. Kondorosi, Legume plant peptides as sources of novel antimicrobial molecules against human pathogens. *Front. Mol. Biosci.* **9**, 870460 (2022).

44. S. Sankari, V. M. P. Babu, K. Bian, A. Alhhazmi, M. C. Andorfer, D. M. Avalos, T. A. Smith, K. Yoon, C. L. Drennan, M. B. Yaffe, S. Lourido, G. C. Walker, A haem-sequestering plant peptide promotes iron uptake in symbiotic bacteria. *Nat. Microbiol.* **7**, 1453–1465 (2022).

45. D. Shen, N. Micic, R. E. Venado, N. Bjarnholt, C. Crocoll, D. P. Persson, S. Samwald, S. Kopriva, P. Westhoff, S. Metzger, U. Neumann, R. T. Nakano, M. Marin Arancibia, T. G. Andersen, Apoplastic barriers are essential for nodule formation and nitrogen fixation in Lotus japonicus. *Science* **387**, 1281–1286 (2025).

46. P.-M. Delaux, S. Schornack, Plant evolution driven by interactions with symbiotic and pathogenic microbes. *Science* **371**, eaba6605 (2021).

47. Y. Lin, Z. Feng, W. Wu, Y. Yang, Y. Zhou, C. Xu, Potential impacts of climate change and adaptation on maize in Northeast China. *Agron. J.* **109**, 1476–1490 (2017).

48. X. Duan, Y. Xie, G. Liu, X. Gao, H. Lu, Field capacity in black soil region, Northeast China. *Chin. Geogr. Sci.* **20**, 406–413 (2010).

49. Y. Bi, H. Zou, C. Zhu, Dynamic monitoring of soil bulk density and infiltration rate during coal mining in sandy land with different vegetation. *Int. J. Coal Sci. Technol.* **1**, 198–206 (2014).

50. J. M. Vincent, *A Manual for the Practical Study of Root-Nodule Bacteria* (Oxford Published for the International Biological Programme by Blackwell Scientific, 1970).

51. J. Schindelin, I. Arganda-Carreras, E. Frise, V. Kaynig, M. Longair, T. Pietzsch, S. Preibisch, C. Rueden, S. Saalfeld, B. Schmid, J. Y. Tinevez, D. J. White, V. Hartenstein, K. Eliceiri, P. Tomancak, A. Cardona, Fiji: An open-source platform for biological-image analysis. *Nat. Methods* **9**, 676–682 (2012).

52. S. Schlüter, A. Sheppard, K. Brown, D. Wildenschild, Image processing of multiphase images obtained via X-ray microtomography: A review. *Water Resour. Res.* **50**, 3615–3639 (2014).

53. Z. Jiang, A. K. Leung, J. Liu, Segmentation uncertainty of vegetated porous media propagates during X-ray CT image-based analysis. *Plant and Soil* **511**, 969–995 (2025).

54. E. S. L. Gastal, M. M. Oliveira, Adaptive manifolds for real-time high-dimensional filtering. *ACM Trans. Graph.* **31**, 1–13 (2012).

55. M. Milatz, E. Andò, G. Viggiani, S. Mora, In situ X-ray CT imaging of transient water retention experiments with cyclic drainage and imbibition. *Open Geomech.* **3**, 1–33 (2022).

56. S. Berg, D. Kutra, T. Kroeger, C. N. Straehle, B. X. Kausler, C. Haubold, M. Schiegg, J. Ales, T. Beier, M. Rudy, K. Eren, J. I. Cervantes, B. Xu, F. Beuttenmueller, A. Wolny, C. Zhang, U. Koethe, F. A. Hamprecht, A. Kreshuk, ilastik: Interactive machine learning for (bio)image analysis. *Nat. Methods* **16**, 1226–1232 (2019).

57. D. Kim, J. M. Paggi, C. Park, C. Bennett, S. L. Salzberg, Graph-based genome alignment and genotyping with HISAT2 and HISAT-genotype. *Nat. Biotechnol.* **37**, 907–915 (2019).

58. S. Anders, P. T. Pyl, W. Huber, HTSeq—A Python framework to work with high-throughput sequencing data. *Bioinformatics* **31**, 166–169 (2015).

59. G. P. Wagner, K. Kin, V. J. Lynch, Measurement of mRNA abundance using RNA-seq data: RPKM measure is inconsistent among samples. *Theory Biosci.* **131**, 281–285 (2012).

60. Gene Ontology Consortium, The Gene Ontology Resource: 20 years and still GOing strong. *Nucleic Acids Res.* **47**, D330–D338 (2019).

61. M. Kanehisa, M. Araki, S. Goto, M. Hattori, M. Hirakawa, M. Itoh, T. Katayama, S. Kawashima, S. Okuda, T. Tokimatsu, Y. Yamanishi, KEGG for linking genomes to life and the environment. *Nucleic Acids Res.* **36**, D480–D484 (2008).

62. P. Danecek, J. K. Bonfield, J. Liddle, J. Marshall, V. Ohan, M. O. Pollard, A. Whitwham, T. Keane, S. A. McCarthy, R. M. Davies, H. Li, Twelve years of SAMtools and BCFtools. *Gigascience* **10**, giab008 (2021).

63. A. R. Quinlan, I. M. Hall, BEDTools: A flexible suite of utilities for comparing genomic features. *Bioinformatics* **26**, 841–842 (2010).

64. S. Fuchs, M. Kucklick, E. Lehmann, A. Beckmann, M. Wilkens, B. Kolte, A. Mustafayeva, T. Ludwig, M. Diwo, J. Wissing, L. Jansch, C. H. Ahrens, Z. Ignatova, S. Engelmann, Towards the characterization of the hidden world of small proteins in Staphylococcus aureus, a proteogenomics approach. *PLOS Genet.* **17**, e1009585 (2021).

65. C. Gehl, R. Waadt, J. Kudla, R. R. Mendel, R. Hansch, New GATEWAY vectors for high throughput analyses of protein-protein interactions by bimolecular fluorescence complementation. *Mol. Plant* **2**, 1051–1058 (2009).

66. A. M. Bolger, M. Lohse, B. Usadel, Trimmomatic: A flexible trimmer for Illumina sequence data. *Bioinformatics* **30**, 2114–2120 (2014).

67. B. Tjaden, De novo assembly of bacterial transcriptomes from RNA-seq data. *Genome Biol.* **16**, 1 (2015).

68. B. Q. Minh, H. A. Schmidt, O. Chernomor, D. Schrempf, M. D. Woodhams, A. von Haeseler, R. Lanfear, IQ-TREE 2: New models and efficient methods for phylogenetic inference in the genomic era. *Mol. Biol. Evol.* **37**, 1530–1534 (2020).

69. C. Camacho, G. Coulouris, V. Avagyan, N. Ma, J. Papadopoulos, K. Bealer, T. L. Madden, BLAST+: Architecture and applications. *BMC Bioinformatics* **10**, 421 (2009).

70. M. I. Love, W. Huber, S. Anders, Moderated estimation of fold change and dispersion for RNA-seq data with DESeq2. *Genome Biol.* **15**, 550 (2014).

71. H. Nielsen, "Practical applications of language models in protein sorting prediction: SignalP 6.0, DeepLoc 2.1, and DeepLocPro 1" in *Large Language Models (LLMs) in Protein Bioinformatics*, D. B. Kc, Ed. (Springer US, 2025), pp. 153–175.

72. J. T. Robinson, H. Thorvaldsdóttir, W. Winckler, M. Guttman, E. S. Lander, G. Getz, J. P. Mesirov, Integrative genomics viewer. *Nat. Biotechnol.* **29**, 24–26 (2011).
